# Regeneration in the Auditory Organ in Cuban and African Dwarf Crocodiles (*Crocodylus rhombifer* and *Osteolaemus tetraspis*) Can We Learn From the Crocodile How to Restore Our Hearing?

**DOI:** 10.3389/fcell.2022.934571

**Published:** 2022-07-04

**Authors:** Hao Li, Karin Staxäng, Monika Hodik, Karl-Gunnar Melkersson, Mathias Rask-Andersen, Helge Rask-Andersen

**Affiliations:** ^1^ Department of Surgical Sciences, Head and Neck Surgery, Section of Otolaryngology, Uppsala University Hospital, Uppsala, Sweden; ^2^ The Rudbeck TEM Laboratory, BioVis Platform, Uppsala University, Uppasala, Swedan; ^3^ Karl-Gunnar Melkersson, Kolmårdens Tropicarium AB, Kolmården, Sweden; ^4^ Department of Immunology, Genetics and Pathology, Science for Life Laboratory, Uppsala University, Uppsala, Sweden

**Keywords:** crocodilian, regeneration, progenitors cells, hair cells, gap junctions

## Abstract

**Background:** In several non-mammalian species, auditory receptors undergo cell renewal after damage. This has raised hope of finding new options to treat human sensorineural deafness. Uncertainty remains as to the triggering mechanisms and whether hair cells are regenerated even under normal conditions. In the present investigation, we explored the auditory organ in the crocodile to validate possible ongoing natural hair cell regeneration.

**Materials and Methods:** Two male Cuban crocodiles (*Crocodylus rhombifer*) and an adult male African Dwarf crocodile (*Osteolaemus tetraspis*) were analyzed using transmission electron microscopy and immunohistochemistry using confocal microscopy. The crocodile ears were fixed in formaldehyde and glutaraldehyde and underwent micro-computed tomography (micro-CT) and 3D reconstruction. The temporal bones were drilled out and decalcified.

**Results:** The crocodile papilla basilaris contained tall (inner) and short (outer) hair cells surrounded by a mosaic of tightly connected supporting cells coupled with gap junctions. Afferent neurons with and without ribbon synapses innervated both hair cell types. Supporting cells occasionally showed signs of trans-differentiation into hair cells. They expressed the MAFA and SOX2 transcription factors. Supporting cells contained organelles that may transfer genetic information between cells, including the efferent nerve fibers during the regeneration process. The tectorial membrane showed signs of being replenished and its architecture being sculpted by extracellular exosome-like proteolysis.

**Discussion:** Crocodilians seem to produce new hair cells during their life span from a range of supporting cells. Imposing efferent nerve fibers may play a role in regeneration and re-innervation of the auditory receptors, possibly triggered by apoptotic signals from wasted hair cells. Intercellular signaling may be accomplished by elaborate gap junction and organelle systems, including neural emperipolesis. Crocodilians seem to restore and sculpt their tectorial membranes throughout their lives.

## 1 Introduction

Birds and their crocodilian relatives rely on hearing for several types of communication. They have a common ancestral origin that also includes the dinosaurs of the Mesozoic period (Gleich and Manley, 2000). Both have a rich vocal repertoire, and their hearing organs, their basilar papillae, share many similarities and neurophysiological properties (Manley, 1970; Caspers, 1984; Gleich and Manley, 2000; Vergne et al., 2009). A major difference is the crocodilians’ adaptation to detecting sound in both air and water.

It is now recognized that auditory receptors in several non-mammalian species can undergo cell renewal and regeneration. Post-embryonic generation of hair cells (HCs) can be induced by trauma, such as noise and aminoglycoside injection, activating cell proliferation and the formation of new HCs (Corwin, 1981a; Cruz et al., 1987; Corwin and Cotanche, 1988; Ryals and Rubel, 1988). This has raised hope of finding new cures for sensorineural deafness in humans by inducing regeneration that is still widely believed to be nonexistent (McLean et al., 2021). In the chick model, new receptors seem to develop from a population of supporting cells (SCs) or progenitor cells, either through mitotic cell division (Girod et al., 1989; Raphael, 1992) and/or direct trans-differentiation, which is defined as HC production without cell mitosis (Adler and Raphael, 1996; Roberson et al., 1996; Benkafadar et al., 2021). Resident SCs in the superior region of the avian basilar papilla and hyaline/cuboidal cells in the inferior part were found to differentiate into new innervated HCs that migrate into the sensory lining (Girod et al., 1989). The triggering signals for this induction, however, remain unknown.

There is still some uncertainty concerning whether SCs and HCs may regenerate even under normal conditions. A production of these cells has been seen in vestibular sensory epithelia of the avian inner ear (Jørgensen and Mathiesen, 1988; Roberson et al., 1992). HCs are produced throughout life in the ears and lateral line organs in bony fishes, lamprey, and amphibians and in the acoustically sensitive macula neglecta of adult sharks (Lowenstein et al., 1968; Corwin, 1981b; Popper and Hoxter, 1984;; Corwin, 1985; Presson and Popper, 1990; Corwin, 1992). A few regenerated SCs and HCs were seen in the normal young adult quail (Ryals and Westbrook, 1990). New supporting border cells (BCs) and hyaline cells (HyCs) were found to be produced postnatally in the normal chicken at the apical half of the basilar papilla (Oesterle and Rubel, 1993). The barn owl maintains physiological thresholds at old age, suggesting there is little loss of receptors and that they are maintained by the production of new HCs (Krumm et al., 2017). Nonetheless, while dividing cells were observed after acoustic damage to the chick auditory epithelium, there were no signs of DNA replication of unexposed chicks (Katayama and Corwin, 1989; Corwin and Warchol, 1991). Proliferation of SCs was limited and mitotic figures were absent, suggesting that there is no cell division during postembryonic life (Corwin and Cotanche, 1988; Jørgensen and Mathiesen, 1988; Girod et al., 1989). Likewise, the pro-neural transcription factor Atoh1, necessary for the development of HCs, was only expressed in the auditory epithelium in damaged birds (Cafaro et al., 2007).

In the present investigation, we explored whether there are signs of normal HC regeneration in the crocodilian papilla basilaris. Our aim was to identify the mechanisms behind it in order to develop a way to restore hearing in man. Two crocodilian family species of different ages were used to study the fine structure of the basilar papilla in the Cuban crocodile (*Crocodylus rhombifer*) and protein/transcription factor expression in the African Dwarf crocodile (*Osteolaemus tetraspis*), including confocal microscopy. There have been relatively few anatomical descriptions of the crocodilians’ auditory organs since Retzius’s classical work (Retzius, 1884; Boord, 1961; Baird, 1974; Leake, 1977; Drenckhahn et al., 1991; Gleich and Manley, 2000). However, a transmission and scanning electron microscopy (TEM and SEM) study was performed in *Caiman crocodilus* (von Düring et al., 1974).

Our findings suggest that crocodile auditory receptors may undergo postembryonic refurbishment without synchronized cell division. HCs seem to develop partially from hyaline HyCs, cubic cells (CCs) and mitochondria-rich cells in the lateral region and a population of SCs beneath the tall hair cells (THCs). From these zones, a step-wise maturation of HCs appears to ensue. Based on the findings, we hypothesize that trans-differentiation and re-innervation may be instigated by efferent nerve fibers via the vestibulocochlear anastomosis (VCA). There seems to be a remarkable renewal of the tectorial membrane (TM), including the formation of ciliary alveolar spaces that appear to be shaped by extracellular exosome-like proteolysis.

## 2 Material and Methods

Two male specimens of the Cuban crocodile (*Crocodylus rhombifer*) with a weight of around 250 g were anesthetized using 5 mg ketamine and 0.05 mg medetomidin and euthanized using an intracardial injection of 0.4 ml T-61. The skull was separated, and the temporal bones were removed using an oscillating saw. The eardrum and the columella were removed, and the ears were immersed in 2.5% glutaraldehyde and 1% paraformaldehyde (PFA) in 2.5% phosphate buffer. The temporal bones were placed in fixative for 48 h and in 0.1 M sodium-ethyl-diamine-tetra-acetic-acid (Na-EDTA) for 3 weeks. Thereafter, the surrounding bone was further removed, and the ears were placed in 1% osmium tetroxide. The specimens were dehydrated in graded ethanol and embedded in Epon. The embedded specimens were divided into different pieces and mounted for semi-thin sectioning. Sections were stained in toluidine blue and photographed. Areas of interest were thin-sectioned, and the sections were stained in lead citrate and uranyl acetate and examined at 80 kV with a Tecnai™ G2 Spirit TEM (Thermo Fisher/FEI Company, Eindhoven, NL). Images were acquired with an ORIUS™ SC200 CCD camera (Gatan Inc., Pleasanton, CA, United States) using Gatan Digital Micrograph software.

### 2.1 Immunohistochemistry

An adult male African Dwarf crocodile (*Osteolaemus tetraspis*), with a bodyweight of 19 kg, was euthanized with 5 ml of pentobarbitalum, 400 mg/ml, injected intracardially 3 h after having been chemically immobilized with a total dose of 300 mg Zoletil^®^ (tiletamin + zolazepam) and 3 mg medetomidine injected i. m. The skull was separated, and the temporal bones were removed using an oscillating saw. The eardrums and the ossicles were removed, and the bones were immersed in 4% PFA in phosphate buffer. The bones were placed in a fixative for 48 h, followed by decalcification in 0.1 M Na-EDTA for 3 weeks. The specimen was dehydrated in graded ethanol and embedded in OCT (Tissue-Tek^®^ O.C.T. stands for optimal cutting temperature) for sectioning followed by incubation with antibodies (Table 1).

**TABLE 1:** Antibodies used in the present investigation.

	Host	Clone	Dilution	Cat #	Company
MAFA	Rabbit	Polyclonal	1:800	ab26405	Abcam
SOX2	Rabbit	Polyclonal	1:400	ab97959	Abcam
Pan-Actin	Mouse	Monoclonal (C4)	1:280	MAB1501	Millipore
SLC26A5	Rabbit	Polyclonal	1:200	SAB4300867	Sigma
Collagen II	Mouse	ll-4C11	1:100	CP18	Calbiochem
Cx26	Mouse	CX-12H10	1:100	13–8,100	Invitrogen
Cx30	Rabbit	Polyclonal	1:50	AP11578PU-N	Acris
Peripherin	Rabbit	Polyclonal	1:200	AB1530	Chemicon
NF-L	Goat	Polyclonal	1:300	sc-12980	Santa Cruz
Parvalbumin	Mouse	PARV-19	1:1600	MAB1572	Millipore
TUJ-1	Mouse	Monoclonal	1:100	sc-58888	Santa Cruz
GRAMD3	Mouse	G-10	1:50	sc-515266	Santa Cruz
C14orf180	Rabbit	Polyclonal	1:50	NBP1-59522	NOVUS
LGR5	Rabbit	Polyclonal	1:200	AP12376PU-N	Acris
CHAT	Rabbit	Polyclonal	1:50	AB5964	Millipore

### 2.2 Micro-Computed Tomography (Micro-CT)

One male specimen of the Cuban crocodile (*C. rhombifer*) with preserved middle ear bones was immersed in 4% PFA in phosphate buffer and underwent micro-CT and 3D reconstruction. The bone was scanned with micro-CT (SkyScan 1176; Bruker, Kontich, Belgium) using the following parameters: source voltage 65 kV, current 385 mA, pixel size 9 mm, filter 1 mm Al, exposure time 1 s, frame averaging 2, and rotation step 0.30°. The projection images were acquired over an angular range of 360°, with an angular step of 0.3°. In the resultant images, the image size was 4,000 × 2,672 pixels, and the pixel size was 9 mm. Projections were reconstructed using NRECON software version 1.7.0.4 (Bruker) based on the Feldkamp algorithm. A volume rendering technique was used to present a 2D projection of a 3D discretely sampled dataset produced by the micro-CT scanner and visualized with the CTvox application (version 3.0; Bruker). Opacity and gray scale values were adjusted to create a realistic 3D view as close to that of the real bones as possible. Geometric measurements were performed, and pictures were taken with the 3D Slicer program (Slicer 4.6; www.slicer.org). The 3D Slicer is an open software platform for medical image informatics, image processing, and 3D visualization (Fedorov et al., 2012).

## 3 Results

**FIGURE 1:**
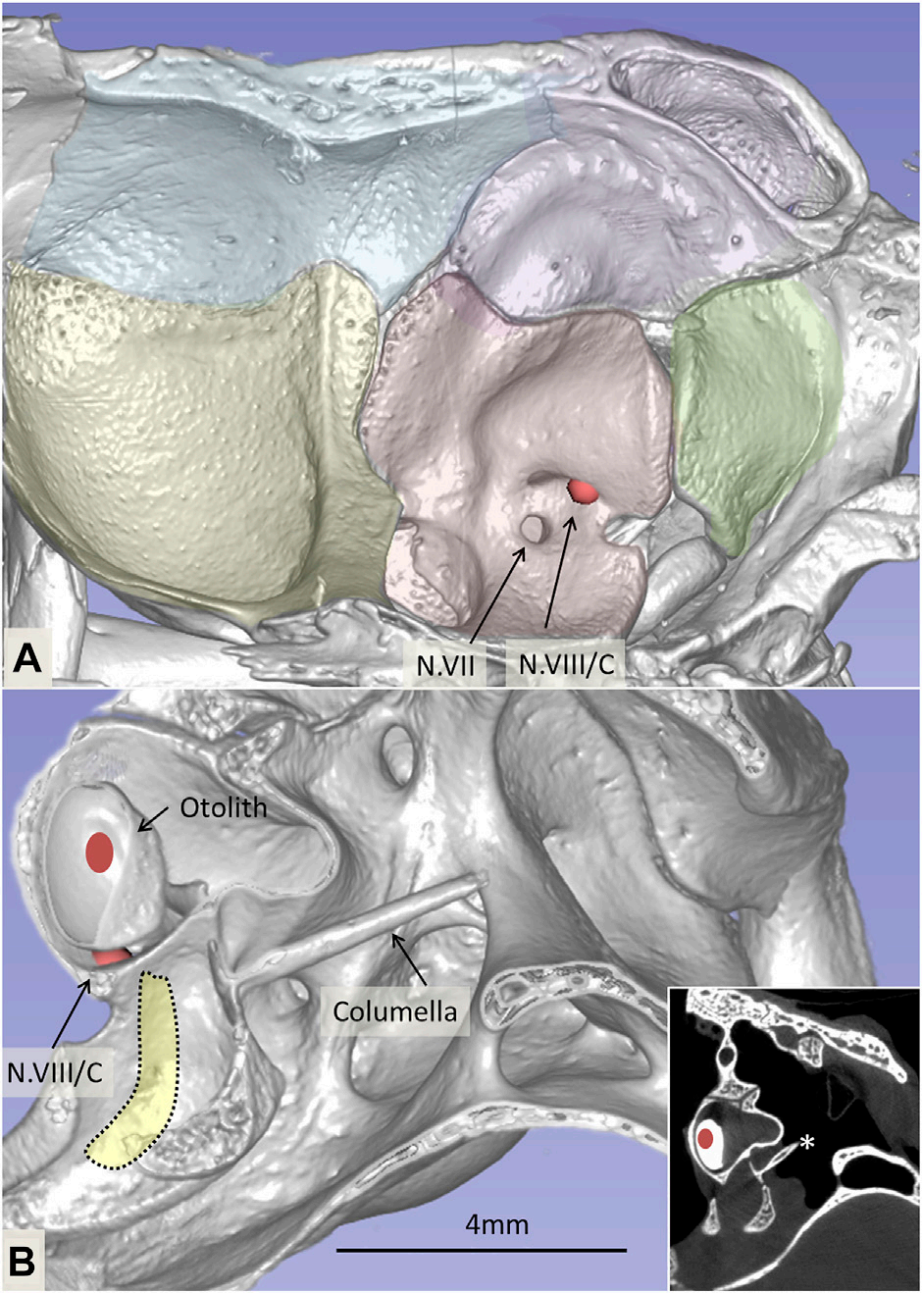
**(A)**. Micro-CT and 3D reconstruction of the right cranial osteology of *C. rhombifer* (medial view). Foramina of the facial (N.VII) and vestibulocochlear cranial nerves (N.VIII) are outlined with arrows. The vestibular and cochlear nerves pass through different foramina. **(B)**. Cropped 3D reconstruction shows the otolith, ear bone columella (*), and the foramen of the cochlear nerve. The inset shows the corresponding coronal section.

Micro-CT and 3D reconstructions showed cranial osteology and localized the foramina of the cochlea-vestibular (N.VIII) nerve and inner ear before dissection (Figure 1). The otolith, the three semi-circular canals, and the position of the basilar papilla were assessed.

### 3.1 Light Microscopy

**FIGURE 2:**
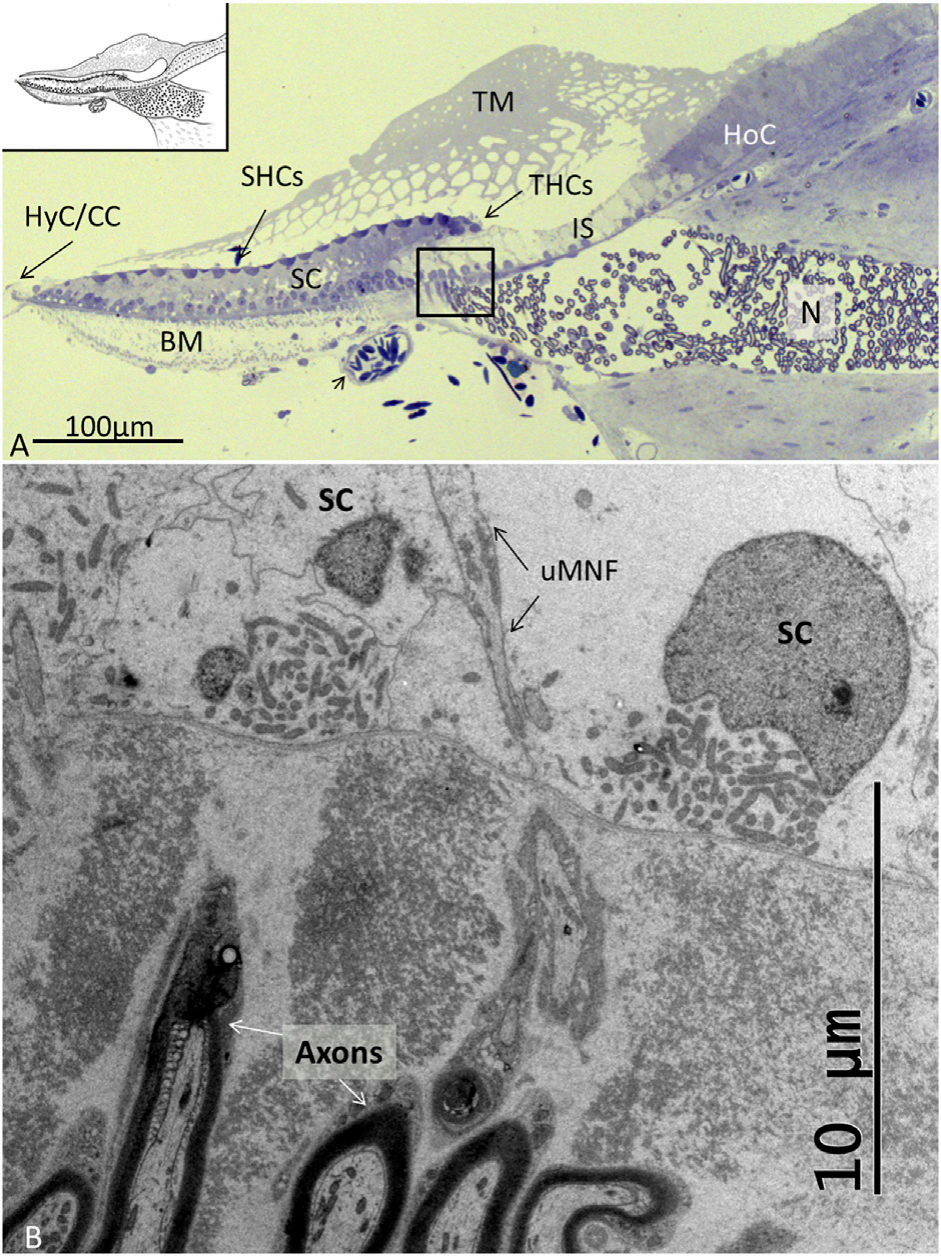
**(A)**. Light microscopy of the papilla basilaris in *C. rhombifer* (high frequency region). The short hair cells (SHCs) and tall hair cells (THCs) are darkly stained with hair tufts reaching into the TM. The auditory organ contains a large population of SCs whose cell nuclei are located basally. The honeycombed TM extends from the HoCs over the inner sulcus (IS). A homogenous population of mostly myelinated nerves (N) enters a fluid-filled space before they reach the habenular openings where they lose the myelin layer. A blood vessel contains nucleated erythrocytes. **(B)**. TEM of the framed area shown in **(A)**. Fixation in 2.5% glutaraldehyde and 1% PFA in 2.5% phosphate buffer. Post-fixation in 1% osmium tetroxide. Toluidine blue staining. BM: basilar membrane, uMNF: unmyelinated nerve fibers, HyC: hyaline cells, cubic cells.

**FIGURE 3:**
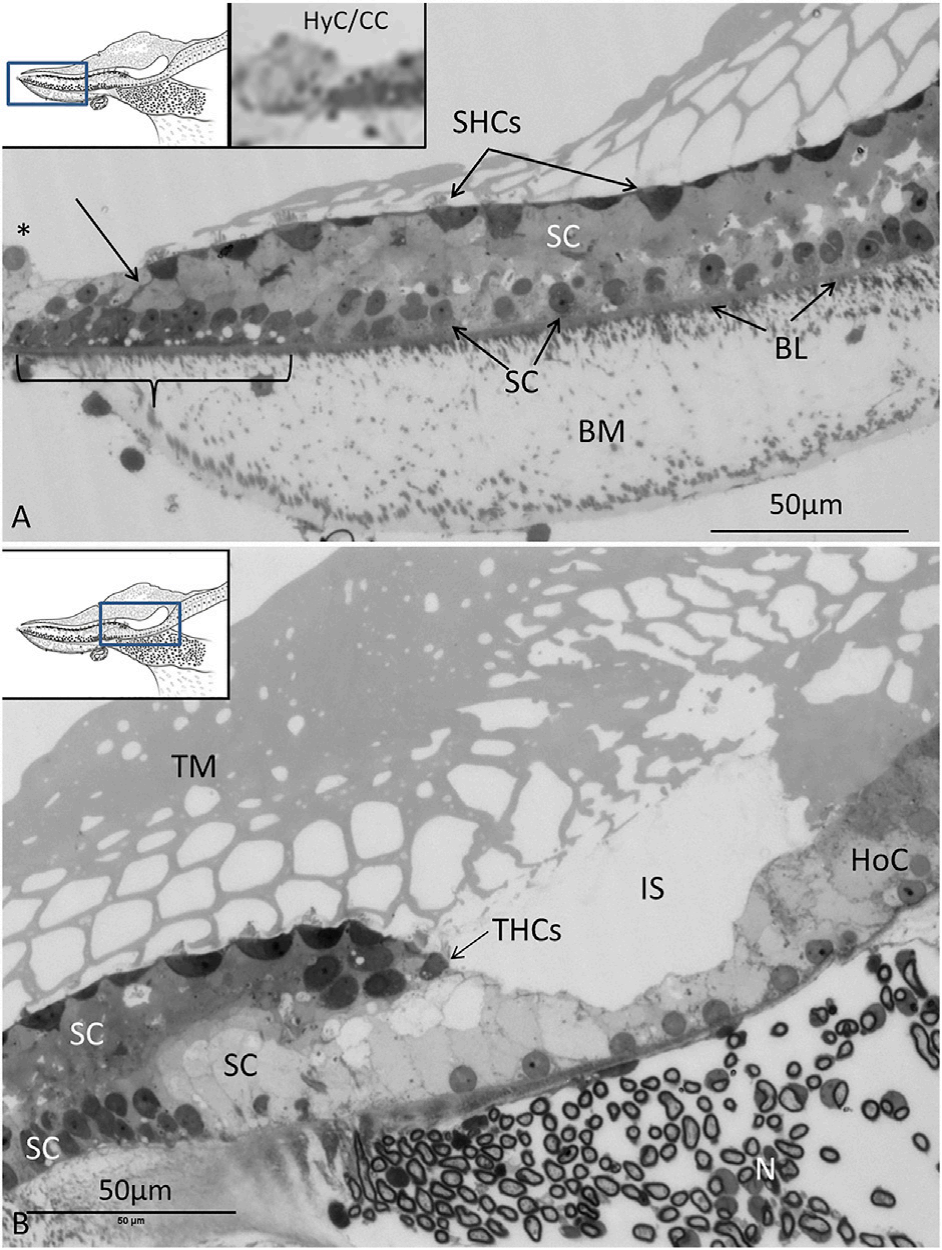
**(A)**. Light microscopy of the abneural region (left inset) of the papilla believed to be a regenerative zone. SCs in this region are often vacuolated (staple), and cell projections connect to the sensory cells (arrow). SC nuclei are basally located. At the border (*), there are HyCs and cubic cells (CCs) that are innervated (right inset). BM: basilar membrane, SHCs: short hair cells, BL: basal lamina. **(B)**. Neural region with THCs and the habenula perforata with entering neurons (N). The TM appears to be secreted from the HoCs with clear endolymph at the inner sulcus (IS).

The auditory papilla basilaris was identified at semi-thin sectioning, and serial thin sections were made at different levels. At cross sections, the organ was wedge-shaped and consisted of a multilayered epithelium resting on a thin basal lamina (Figures 2, 3). Superiorly, a single layer of densely stained HCs was recognized. They were surrounded by a mosaic of supporting SCs. In some regions, the mosaic was less well developed. Few SC nuclei were located at the level of the HCs, but were instead placed basally near the basal lamina. The epithelium rested on a basilar membrane (BM) consisting of radially and longitudinally arranged collagen fibrils with a convexity facing the scala tympani. A spiral vessel was typically located near the habenular opening for the nerve fibers. The TM contained clear spaces or honeycomb-like alveoli. These spaces were formed near the homogene cells (HoCs) above the auditory nerve and enlarged against the sensory cell surface, where the ciliary tufts entered individual spaces.

### 3.2 Transmission Electron Microscopy

#### 3.2.1 Two Types of Sensory Hair Cells: Tall (Inner) and Short (Outer)

**FIGURE 4:**
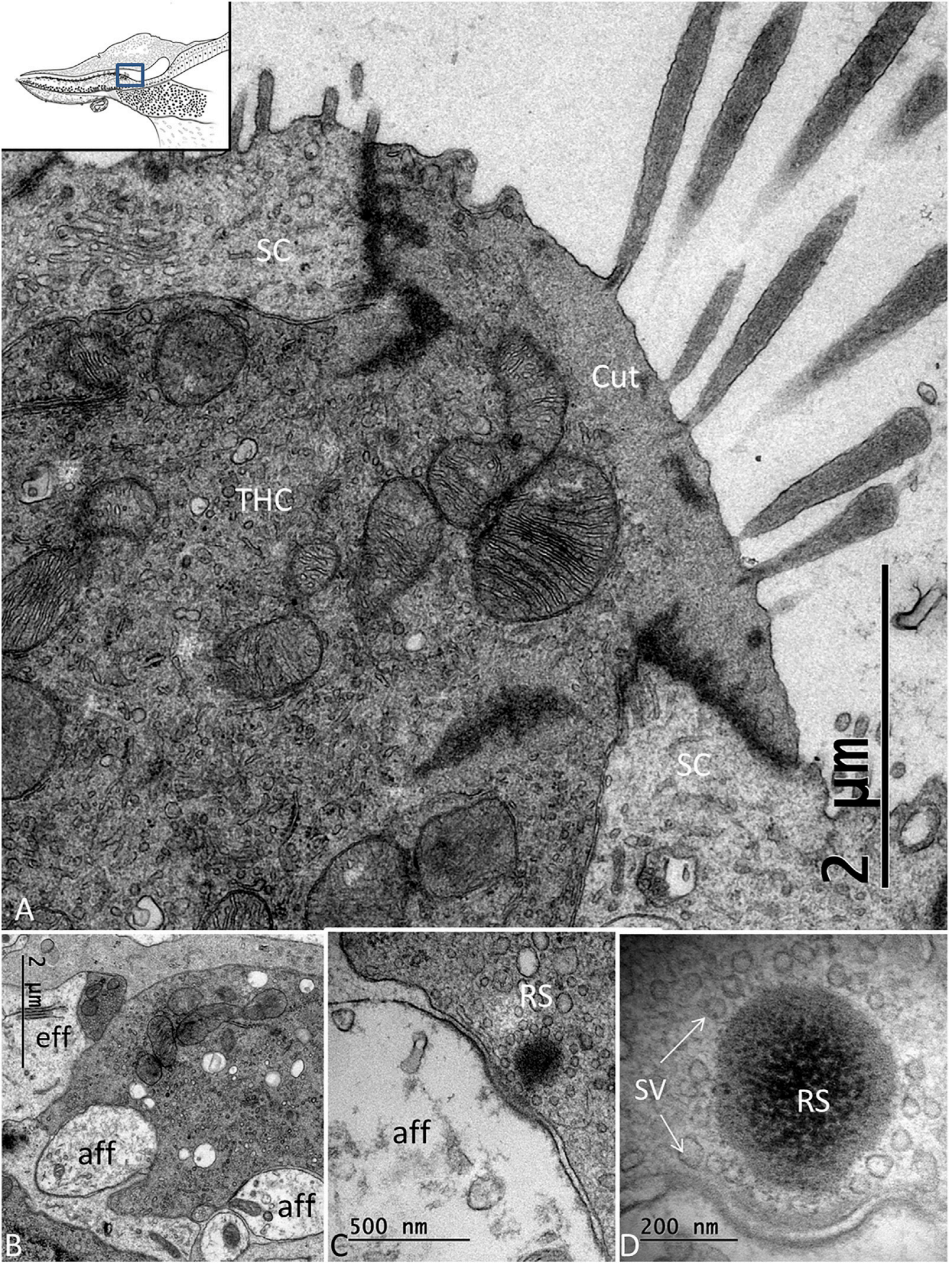
**(A)**. TEM of a THC in the *C. rhombifer*. It is surrounded by two SCs. Stereocilia are anchored in the cuticula (Cut). **(B)**. Several afferents and an efferent terminal innervate the hair cells. **(C)**. An RS is surrounded by a halo of clear synaptic vesicles (SVs). A dense postsynaptic membrane is seen. **(D)**. Higher magnification of an RS with a large dense body surrounded by SVs.

There were two to three rows of THCs, depending on the frequency location at the neural side of the papilla. The THCs were cubic or columnar, had a dark cytoplasm, and were rich in mitochondria (Figure 4). They contained large, clear vesicles of variable sizes and a prominent rough endoplasmic reticulum. The cuticula with stereocilia was centrally placed and contained over 100 stereocilia per cell. A kinocilium was seldom seen. The THCs were connected to both the efferent and afferent nerves’ terminals, often located near each other (Figure 4B). Ribbon synapses (RSs) consisted of large (400 nm) dense bodies surrounded by a halo of clear vesicles (Figures 4B, D). The afferent terminals were electron-lucent and faced ribbon and non-ribbon specializations with increased thickness of the pre- and postsynaptic membranes. The efferent terminals contained abundant clear vesicles, some dense-core vesicles (DCVs), and a large number of mitochondria. The cilia entered the honeycomb spaces and abutted the organic matrix of the TM, where dense specializations of the membrane appeared.

**FIGURE 5:**
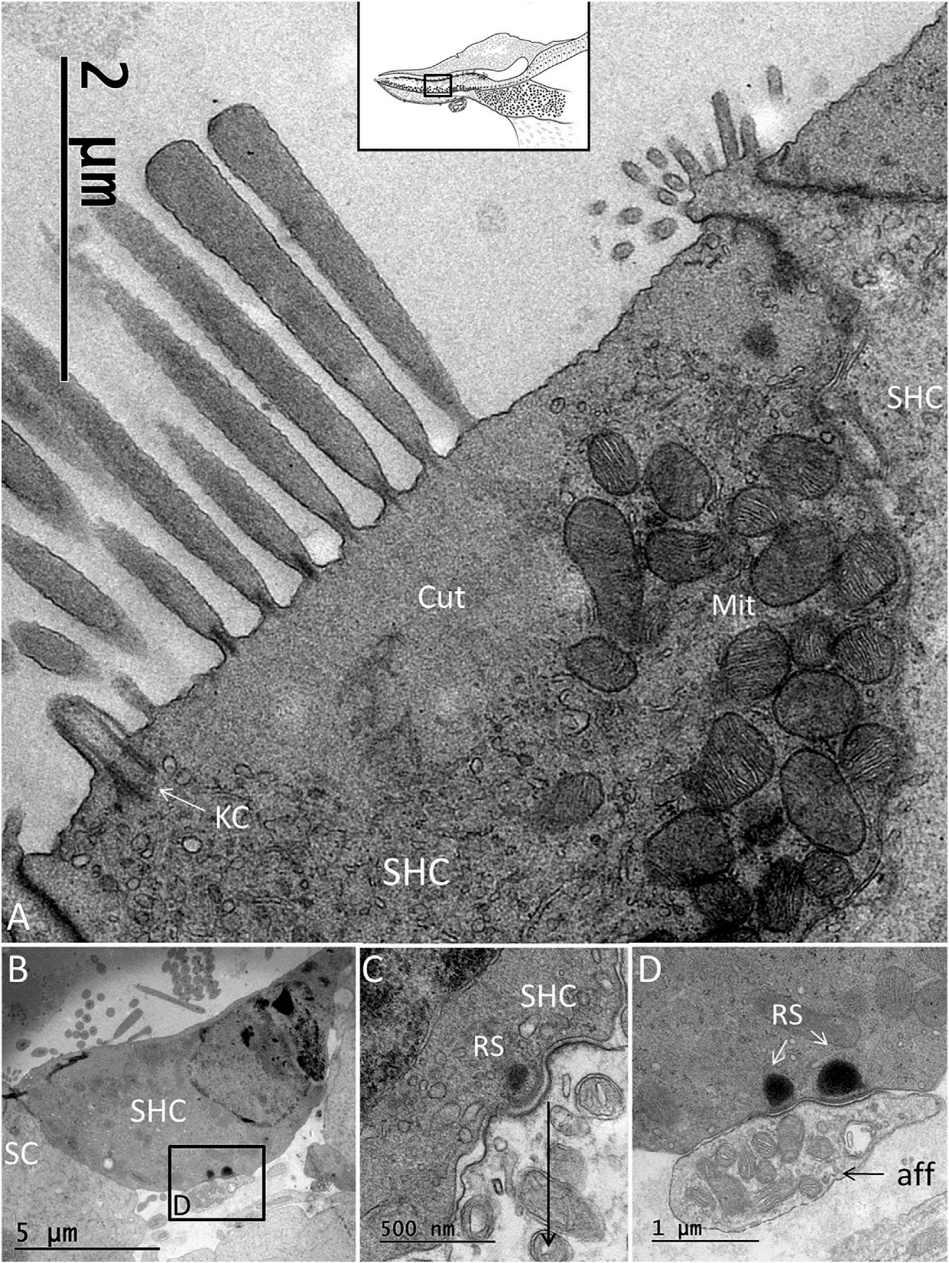
**(A)**. TEM of an SHC of the *C. rhombifer*. **(B)**. One afferent terminal is associated with RSs (framed area magnified in D. **(C)**. SHC with an RS. There is no halo of synaptic vesicles. **(D)**. Framed area in B is magnified and shows two RSs at the afferent (aff) terminal.

**FIGURE 6:**
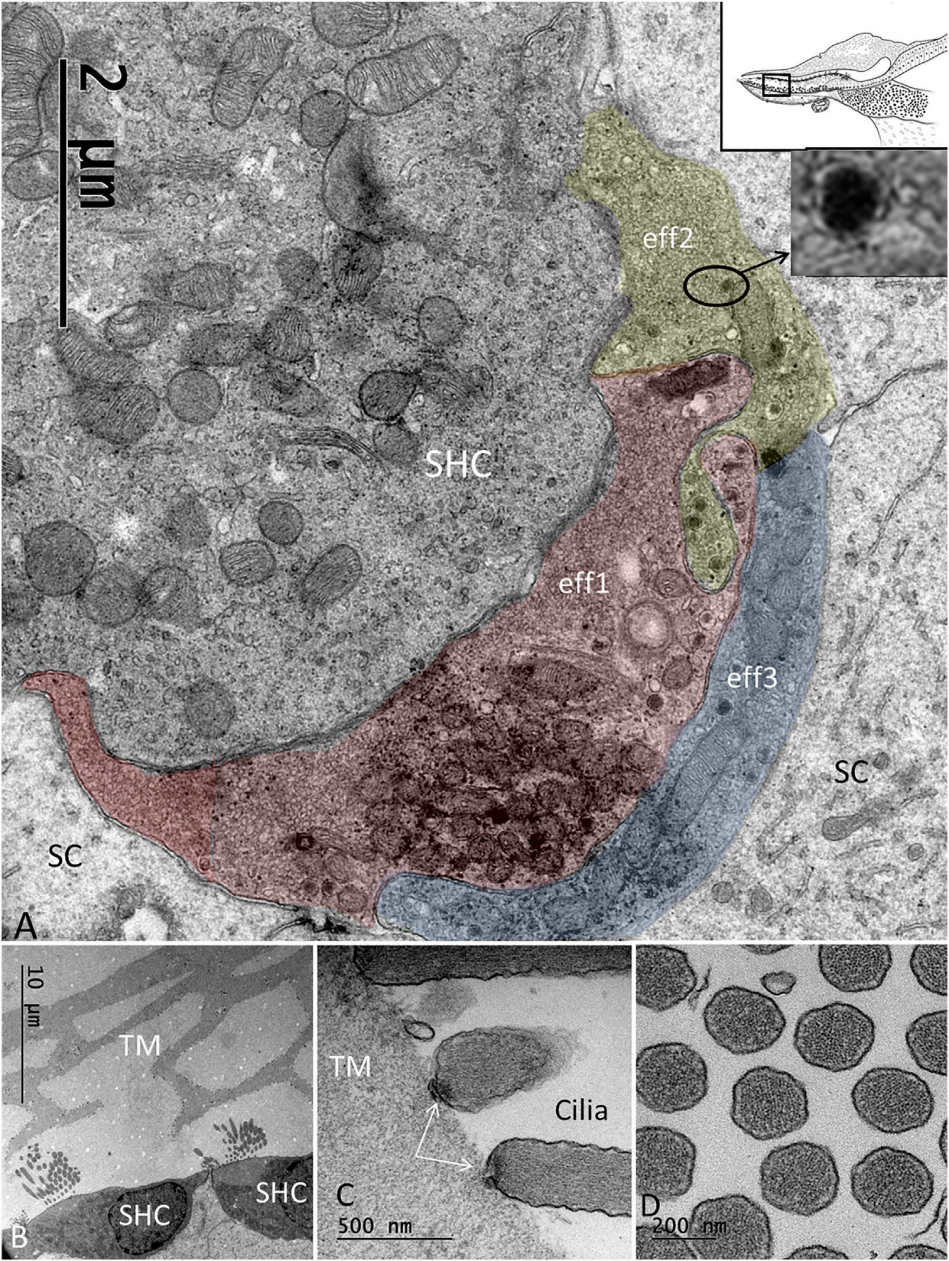
**(A)**. Basal pole of an SHC connected to three efferent terminals (eff1, eff2, eff3). The boutons are filled with synaptic vesicles and abundant mitochondria. **(B)**. SHCs and associated TM having a honeycomb structure. **(C)**. The ciliary tips of the SHCs show specializations at the contacts points with the TM. **(D)**. Cross-sectioned stereocilia of an SHC show abundant actin filaments.

**FIGURE 7:**
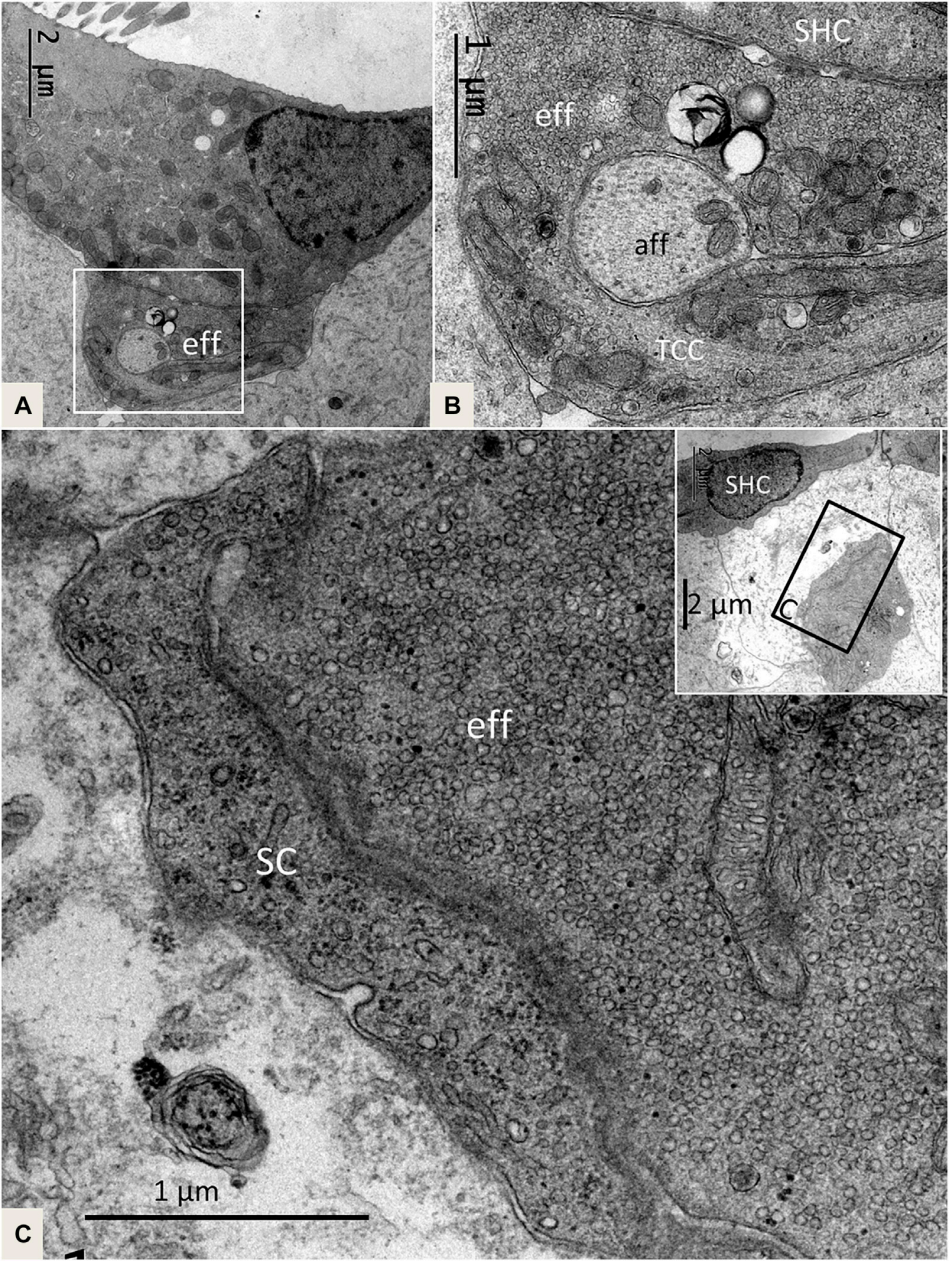
**(A)**. SHC with an efferent terminal rich in secretory vesicles. Framed area is magnified in **(B)**. **(B)**. The efferent terminal has folded and enwraps an afferent fiber in a so-called emperipolesis. The terminal contains micro-tubules or a transcellular channel system (TCC). Eff: efferent nerve fiber, aff: afferent nerve fiber. **(C)** and inset. A large efferent swelling is located freely in the extracellular tissue beneath an SHC. It is associated with an electron-dense SC.

The SHCs were cup-shaped and either flat or cubic (Figure 5A). Some had no ciliary tufts and looked immature. The stereocilia and cuticle were placed laterally and contained actin filaments with rootlets entering the cuticular plate. The SHCs were also reached by lucent afferent terminals and darkly stained efferent nerve terminals. Afferent RSs with large, dense bodies were surrounded by a halo of clear synaptic vesicles. At some terminals, there were two associated RSs (Figures 5B–D). There were also RSs that lacked contact with a nerve terminal. Some RSs lacked the halo of clear vesicles. Occasionally, several entwined efferent terminal swellings embraced the entire basal pole of the SHC (Figure 6A). They contained large numbers of clear synaptic and dense-core vesicles. The postsynaptic membrane showed a sub-membrane cisternae. The membrane tips of the longest cilia showed electron-dense specializations projecting into the TM (Figures 6B–D). Some efferent terminals folded around afferent nerve fibers in a so-called emperipolesis (Figures 7A,B). Occasionally, large efferent swellings lay independently beneath the SHCs connected to electron-dense SCs (Figure 7C).

#### 3.2.2 Supporting Cells (SCs)

**FIGURE 8:**
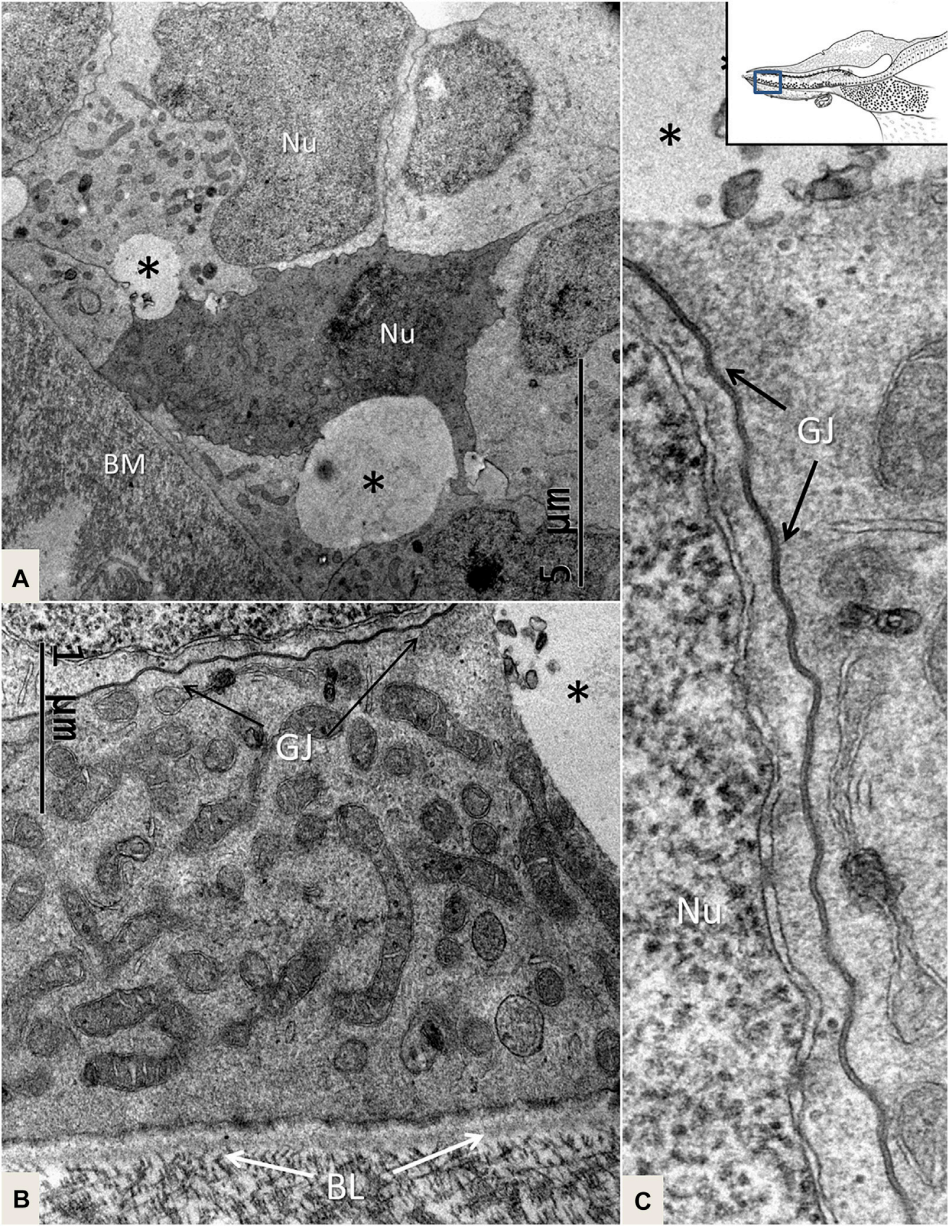
TEM of mitochondria-rich SCs near the BM. One cell is more electron-dense, and there are clear intercellular vesicles (*). **(B)**. SCs display prominent GJs. **(C)**. Higher magnification of an intercellular GJ shown in **(B)**. BL: basal lamina, Nu: nucleus.

The papilla basilaris mostly contained a tightly arranged mosaic of electron-lucent SCs extending from the BM to the endolymph surface. Superiorly, SCs surrounded the HCs and were more organelle-rich with prominent Golgi apparatuses, smooth endoplasmic reticuli, and small vesicles. The extracellular space was minimal, and we found that the SCs displayed prominent intercellular gap junctions (GJs), especially basally. One or two layers of cell nuclei were located basally, often as a “string of pearls,” as shown in the chicken papilla. The basal SCs contained many mitochondria located against the basal lamina (Figure 8). Apically, the SCs formed tight junctions, and finger-like microvilli protruded into the endolymph and cytoplasmic blebs. The SCs were surrounded by many unmyelinated nerve fibers. No mitoses were seen, but some nuclei lacked a complete nuclear coat and filled up the cell almost completely.

#### 3.2.3 Nerve Supply of the Papilla Basilaris

The auditory nerve fibers were mostly myelinated with a uniform size and a diameter of around 4 μm. The axoplasm diameter was around 2 μm. Thin unmyelinated nerve fibers were spread among the myelinated axons. They had a diameter of around 0.25–1.0 μm, and several were enwrapped by a single Schwann cell. They contained 20 nm thick neuro-tubules and mitochondria. The auditory nerve fibers entered a fluid-filled space before reaching the papilla basilaris. This space was lined with a mesothelial layer. Just before nerve fibers perforated the basal lamina, they lost myelin and were gated into the papilla surrounded by collagen pillars (Figure 2B). Some axons radiated directly to the THCs, but also spread basally among SCs, HCs, HyCs, and CCs.

As earlier described, efferent cochlear fibers traverse the superior vestibular ganglion and enter the inferior vestibular nerve (Boord, 1961). From there, they separate into a fascicle known as the VCA. It extends from the saccular ganglion (SG) to the cochlear nerve and consists of predominantly efferent fibers. Efferent nerves course along the cochlear ganglion (CG) border, and fibers left along afferent radial fibers. They reach the receptor epithelium, but their ultimate termination could not be determined in his light microscopic study. In the present study, the SG cell Soma in the *C. rhombifer* typically varied in size compared with that of the more homogenously sized CG cells. The VCA was localized at the separation between the SG and CG (Supplementary Image S1A). The nerve bundle consisted of myelinated fibers with different diameters. These nerves followed the afferent fibers, and before reaching the habenula, they lost myelin, and several axons were embedded in the same Schwann cell (Supplementary Image S1B). These fibers were of a small size (0.25–0.5 µm) and typically contained DCVs (inset, Supplementary Image S1B).

#### 3.2.4 Hyaline Cells (HyCs) and Cubic Cells (CCs)

**FIGURE 9:**
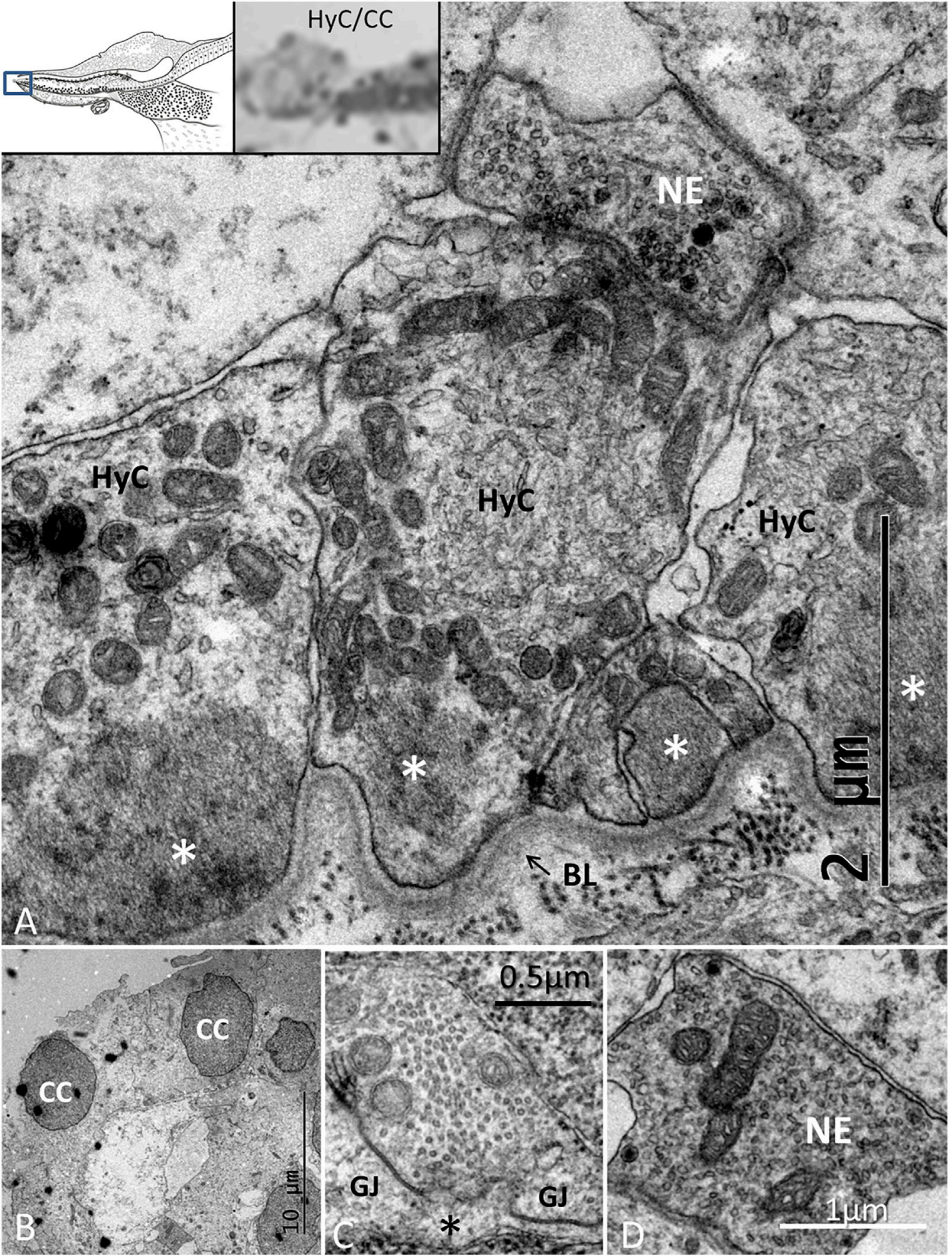
TEM of the HyC/CC region. **(A)**. HyCs show basal fibrillary structures. A basal lamina separates the cells from the BM. A nerve efferent (NE) connects to the HyC. The efferent fiber contains an abundance of clear secretory vesicles (inset) and DCVs. **(B)**. CCs with an intercellular vacuole. **(C)**. One cell contains micro-tubules near the plasma cell membrane margined by GJ complexes. **(D)**. NE associated with an HyC.

The lateral transitional epithelium between the papilla basilaris and tegmentum vasculosum (TV) contained electron-lucent CCs and HyCs surrounded by nerve fibers (Figure 9). The HyCs were rich in mitochondria and contained bundles of actin fibrils facing the basal lamina. The cells were associated with many nerve fibers, predominantly efferents rich in clear synaptic and dense-core vesicles (Figure 9 and Supplementary image 2). Synapse-like junctions were noted against the epithelial cells. Some cells were more electron-dense and irregular, and they interacted physically with efferent swellings, also reaching the SHCs. The cells were connected through GJs. These cells were rich in micro-tubular arrangements, here referred to as the transcellular channels (TCCs).

#### 3.2.5 Tectorial Membrane (TM) and Homogene Cells (HoCs)

**FIGURE 10:**
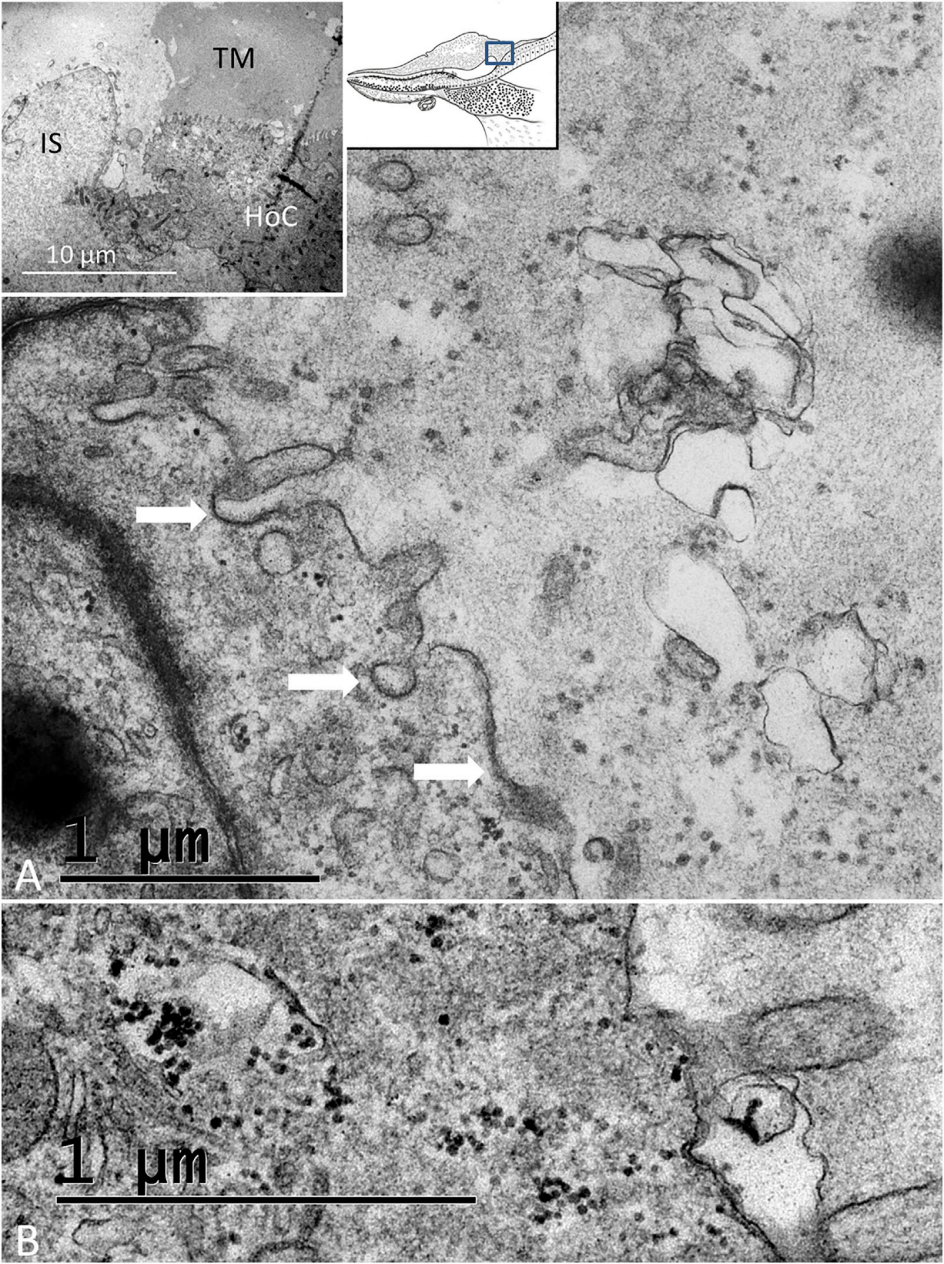
**(A)**. Electron microscopy of an HoC showing secretory activity. Several coated vesicles and pits (arrows) are seen at the apical cell surface. The TM contains apoptotic vesicles and electron-dense granules. Inset shows the transition zone between the HoC and IS. Microvesicles aggregate in the apical cytoplasm (white arrow) and empty their content of granules into the lumen (dark arrow). **(B)**. Dense particles in the apical cytoplasm of a hyaline cell and in the lumen.

**FIGURE 11:**
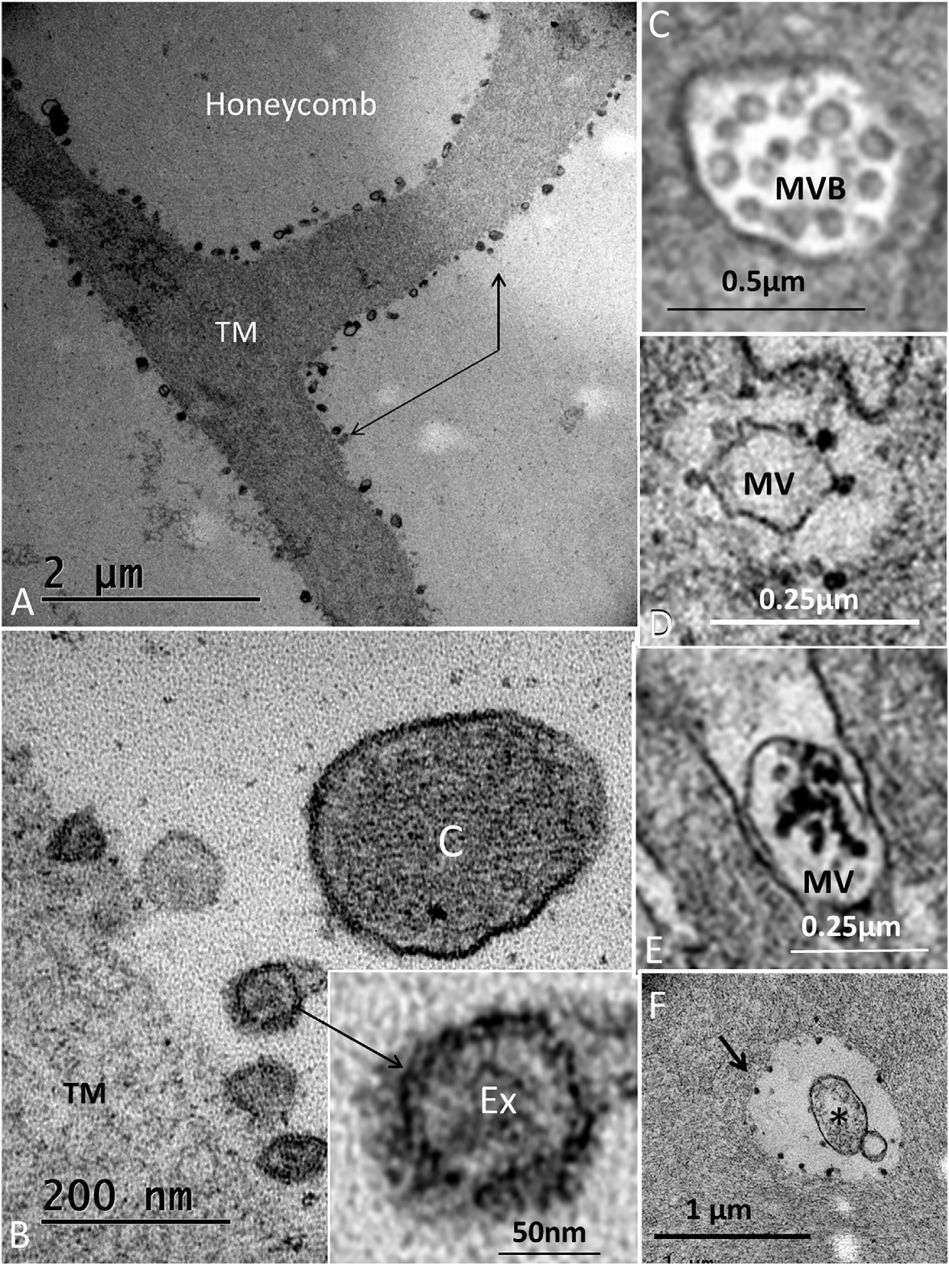
**(A)**. Electron microscopy of the honeycomb layer near the sensory cells. The beams are outlined by hollow electron-dense granules (indicated by arrows). **(B)**. Higher magnification shows membrane-bound exosome-like bodies (Ex) further magnified in the inset. **(C)** cilium. **(C)**. MVB located in the HoC. **(D)**. Microvesicle in the apical cytoplasm of an HoC is surrounded by electron-dense bodies. **(E)**. Microvesicle expelled in the endolymph located between apical projections containing electron-dense bodies. **(F)**. Similar bodies outline a vacuole in the TM (arrow). *: apoptotic microvesicle.

The wedge-shaped TM consisted of an extracellular matrix, whose thickness decreased gradually from the HoCs to the lateral region of the papilla. The TM contained vacuolar spaces, resulting in a honeycomb-like appearance (Figure 2B; Figure 5B). These clear spaces, or alveoli, increased in size against the HCs whose ciliary tufts were housed in hollow spaces. The HoCs showed signs of merocrine and apocrine secretion into the TM with a sharp demarcation zone against the clear cells at the inner sulcus (IS) (Figure 10). This seemed to indicate that the HoCs secreted the matrix components of the TM. The apical cell membrane displayed finger-like projections that were anchored deep in the cytoplasm (Supplementary Image S3). The cytoplasm contained multivesicular bodies (MVBs) and microvesicles that contained electron-dense granules (Figure 10). These granules were found in the TM matrix and seemed to be secreted or expelled from the HoCs. In all investigated ears, the TM contained membrane-bound exosome-like figures with a diameter of around 50–100 nm. They seemed to be secreted or shed into the extra-cellular space (Figure 10B). They had a lytic influence on the extracellular matrix, and so appeared to sculpt the honeycomb alveoli through enzymatic digestion (Figure 11, Supplementary Image S4).

### 3.3 Immunohistochemistry of the Papilla Basilaris

**FIGURE 12:**
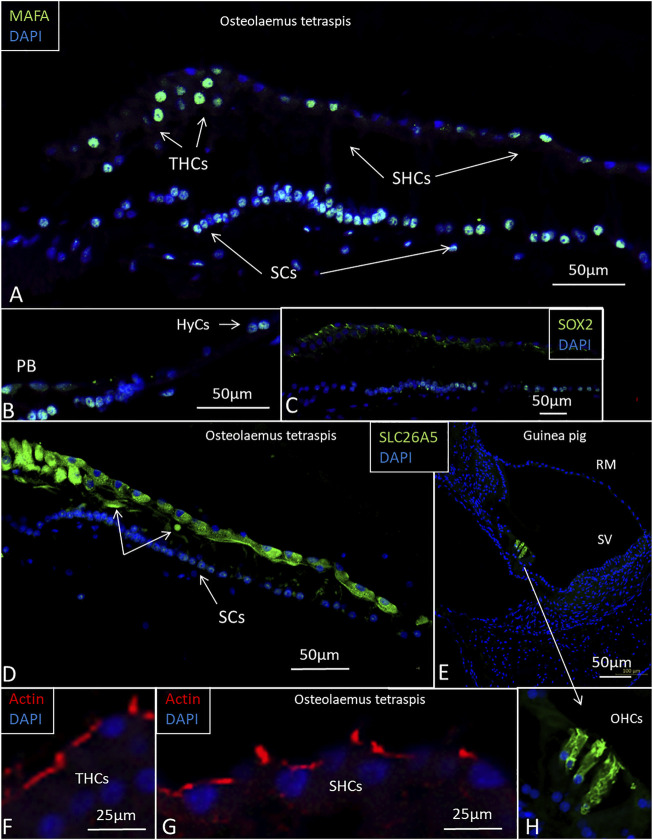
Imunofluorescence of the papilla basilaris in the *O. tetraspis.* **(A)**. Some THCs and SHCs show strong expression of MAFA. A few THCs have not reached the epithelial surface. Many SC nuclei express the transcription factor MAFA. **(B)**. Some HyCs express MAFA. **(C)**. Transcription factor SOX2 is expressed in SCs. **(D)**. Sensory cells, including the THCs, express SLC26A5/prestin. **(E)**. Positive control shows SLC26A5/prestin expression in outer hair cells in the guinea pig cochlea. **(F)**. and **(G)**. Actin expression in the stereocilia and cuticular plate of the HCs. **(H)**. Higher magnification of outer hair cells shown in **(E)**.

SHC and THC nuclei expressed the transcription factor MAFA, and several SCs expressed both SOX2 and MAFA (Figures 12A–C). Some HyCs also expressed MAFA. The transcription factor SOX2 was not expressed in the HCs (Figure 12C). The tissue capsule surrounding the inner ear expressed collagen type II. SLC26A5/prestin antibody labeling showed diffuse positive staining of all HCs. A few cells located just beneath the sensory cells also expressed prestin (Figures 12D, H). Positive control staining of the guinea pig cochlea showed intense staining of outer HCs, but not inner HCs (Figures 12E, H). Actin antibodies labeled stereocilia and the cuticular plate of the HCs (Figures 12F, G). Cx26, Cx30, and the THC and SHC markers GRAMD3 and C14ORF180 (Benkafadar et al., 2021) gave negative results. Parvalbumin stained the crocodile THCs and most of the SHCs. Some SHCs did not express parvalbumin, and some HyCs were also positive. Guinea pig controls showed selective staining of the spiral ganglion cells and inner HCs with nerve terminals. The biomarker for Wnt-driven adult stem cells in certain tissues Lgr5 protein (Leucine-rich repeat-containing G-protein-coupled receptor 5) was tested, but gave uncertain results as well as acetylcholinesterase.

### 3.4 Regenerative Domains

**FIGURE 13:**
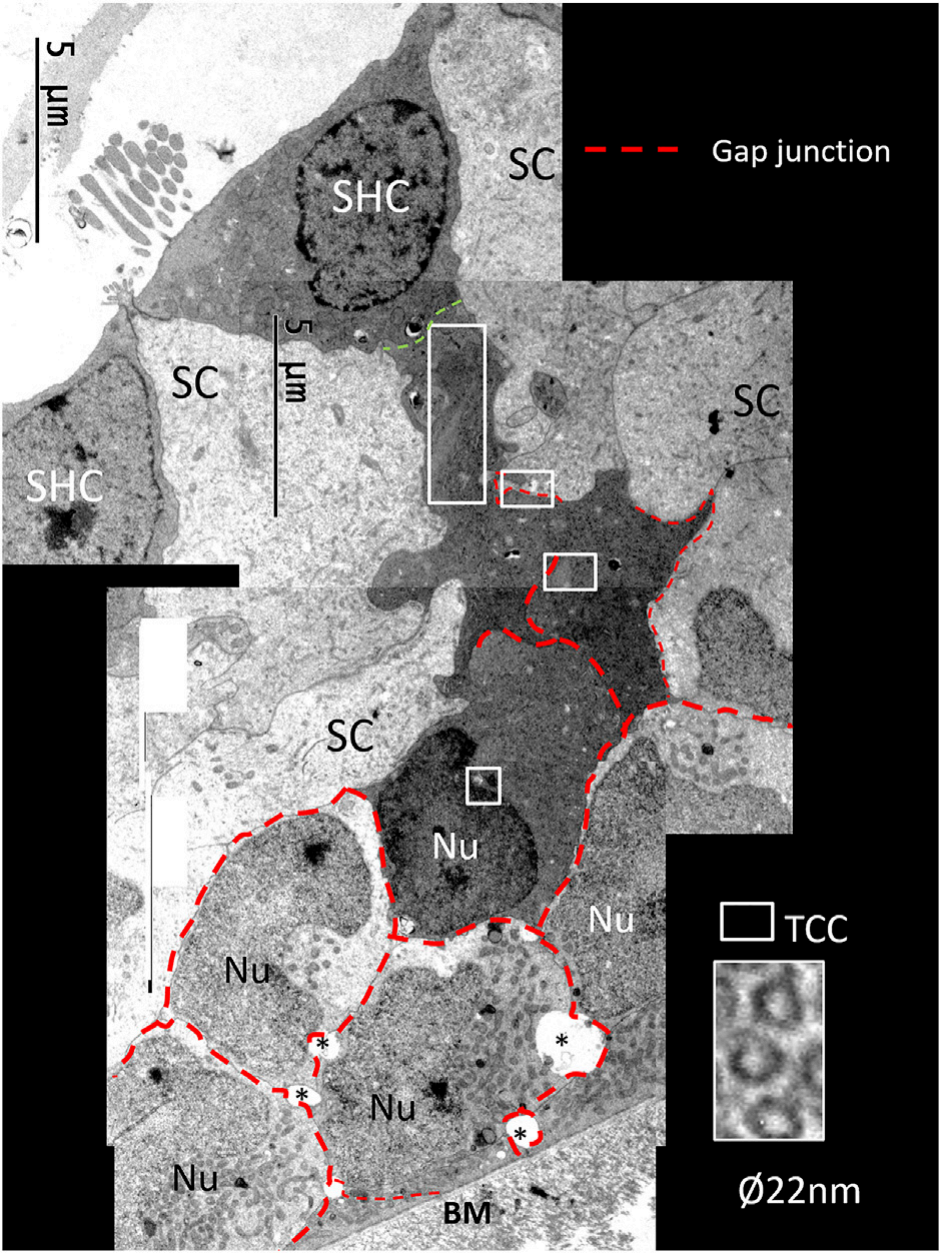
Composite electron micrograph of SCs at the abneural zone of the basilar papilla at the lateral tip of the TM. An SHC is connected to several electron-dense SCs. These cells form a domain joined by GJs (red broken lines). Framed areas show regions with TCCs shown in higher magnifications in Figure 15.

**FIGURE 14:**
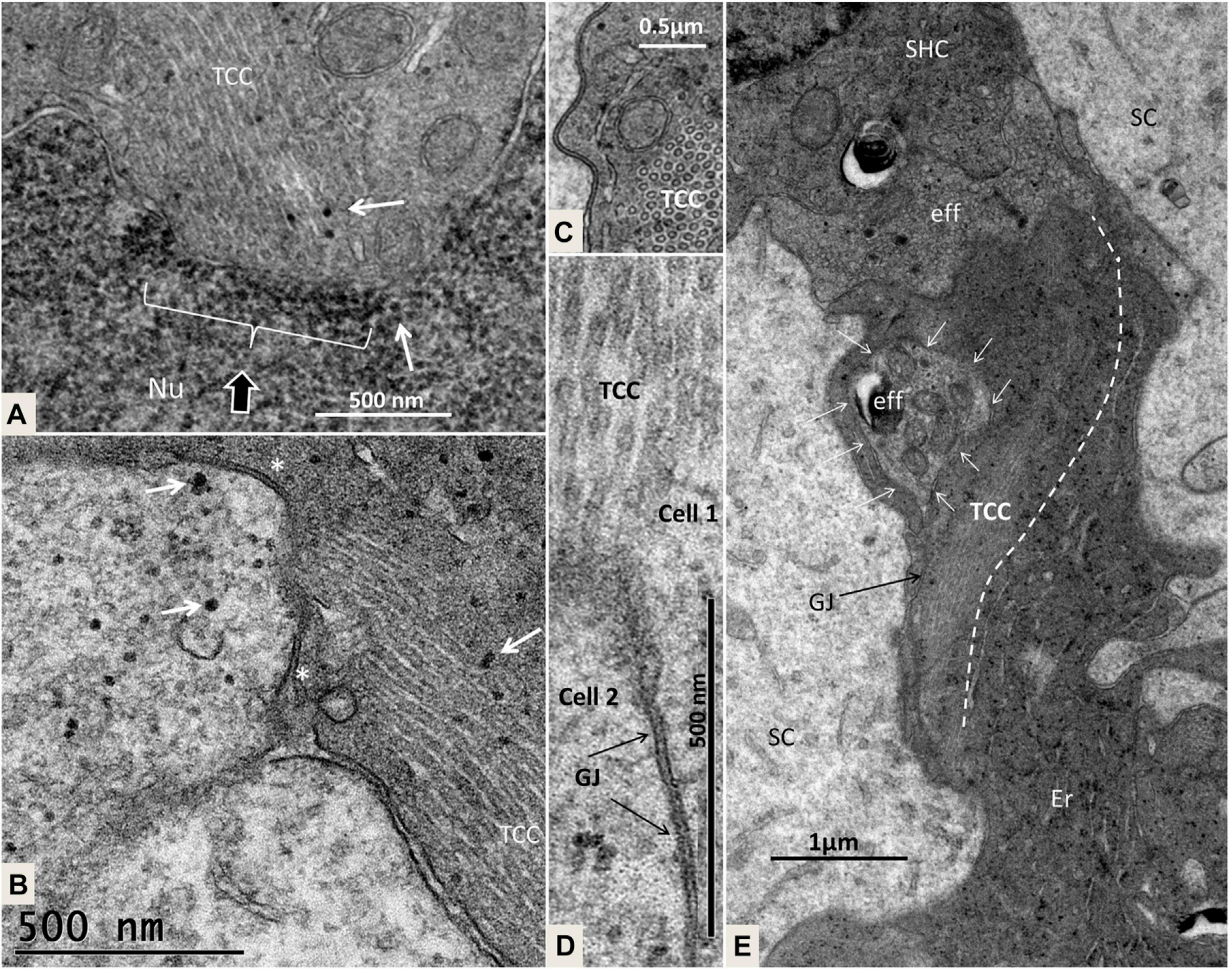
**(A)**. Higher magnification of the framed area shown in Figure 13A. Micro-tubular system (TCC) is connected to the cell nucleus (Nu) in the basal SC. **(B)**. The TCC connects to the cell membrane between 2 GJs (*) to another SC. Micro-tubules contain ribosome-like bodies (arrows) also seen in the adjacent cell. **(C)**. Cross-sectioned TCC with a crystalloid profile. **(D)**. Higher magnification of TCC and associated plasma membrane at the GJ complex (scale bar is 500 nm). **(E)**. Cell complex beneath mature SHCs. Efferent nerve fibers (eff) are seen at the basal pole of the SHC. The electron-dense cells beneath it show an intimate relationship with the efferent nerves, of which the lower nerve fiber shows emperipolesis (white arrows). The TCC runs across the cell to the efferent nerve terminal.

At the abneural papilla, electron-dense SCs occasionally formed cell territories that reached from the basal lamina to the sensory cells (Figure 3A; Figure 13). These enclaves seemed to be involved in the regeneration of HCs and their innervation. The intercellular spaces were sealed by complete GJs that were separated at only a few secretory-like vesicles (Figure 8A; Figure 13). The basal SC nuclei showed prominent nucleoli. The varying texture of chromatin and the poorly defined nuclear coats suggested that they may recently have undergone cell division. The cells were closely associated with efferent nerve fibers. No GJ plaques were found on the HCs or neurons. The SCs contained micro-tubule-like TCCs having a diameter of around 20 nm (Figure 14). They formed straight or undulating pathways between the cell nucleus and plasma membrane (Figures 14A–C). Higher magnification of the TCC complexes suggested that they were related to intercellular communication and to the GJs (Figure 14D). Occasionally, the TCCs contained ribosome-like material, suggesting that they may transfer genetic material between neighboring cells. The intimate relationship between efferent nerve fibers (emperipolesis) and associated electron-dense SCs is demonstrated in Figures 7A, B:Figure 14E.

## 4 Discussion

### 4.1 Crocodile HCs May Undergo Postembryonic Regeneration

Our results suggest that new auditory receptors may arise normally in the crocodile acoustic organ from a population of migrating and trans-differentiating HyCs, CCs, and mitochondria-rich supporting cells. Several SHCs looked immature, were flat, and lacked stereovilli and complete synaptic complexes. Many SHCs, THCs, and SCs expressed MAFA, and some SCs also co-expressed SOX2. MAFA is a transcription factor that can modulate somatic cells and pluripotent stem cells through the regulation of gene expression and trans-differentiation. MAFA factors may regulate tissue-specific gene expression and are involved in cell transformation. Increased expression of MAFA is known to induce sustained proliferation of post-mitotic avian neural retina cells (Benkhelifa et al., 1998). All HCs expressed SLC26A5/prestin suggesting cochlear amplification, and a few cells located beneath the HCs also occasionally expressed prestin (Figure 12D). Hence, THCs and SHCs do not seem to share the separate traits of inner and outer HCs in mammals where only the outer HCs express prestin. Transcriptomic analysis has shown that there is limited molecular homology between avian and mammalian cochlear HC subtypes (Janesick et al., 2021), suggesting that organization of hearing organs may have evolved independently through convergent evolution (Köppl, 2011).

#### 4.1.1 Innervation of SCs—A First Step in HC Regeneration?

The avian and spectacled caiman crocodile auditory organs harbor HyCs that contain contractile proteins. These cells have smooth and polarized muscle-like filament bundles within their basal pole that express actin, myosin, and alpha-actinin (Drenckhahn et al., 1991). These cells were thought to actively modify radial stiffness of the basilar membrane, influencing frequency tuning of primary auditory afferents, which were also shown to be temperature-dependent. There are nerve fibers located around the HyCs and CCs. Some may be directed against the macula lagenae, a vestibular receptor organ (Boord, 1961). The presence of synaptic contacts between efferent nerve fibers and HyCs suggested there is neural control (Drenckhahn et al., 1991; Cotanche et al., 1992; Oesterle et al., 1992). “En passent” efferent synapses with HCs, SCs, and HyCs were earlier described in the *C. crocodilus* as containing clear synaptic vesicles and presynaptic densities (Drenckhahn et al., 1991; von Düring et al., 1974). In the avian, HyCs nerve fibers have terminal swellings rich in clear and dense-core vesicles (Girod et al., 1989; Cotanche et al., 1992; Oesterle and Rubel, 1993; Janesick and Heller, 2019). Efferent innervation without synaptic contacts but expressing acetylcholinesterase was shown in the pigeon (Takasaka and Smith 1971). HyCs were also found to be highly specialized with GJs and pre- and postsynaptic specializations (Oesterle et al., 1992). We found many associated nerve fibers, including contacting efferent nerves, among the HyCs but also electron-dense SCs without synaptic specializations, such as subsurface cisternae. The electro-dense GJ-coupled SCs formed enclaves extending to the sensory epithelium. SCs were tightly connected to neurons (peripolesis), but occasionally showed emperipolesis with neurons entirely engulfed by the SC, suggesting both a molecular intercellular communication and mechanical influence. This is in accordance with earlier descriptions of the regenerative capacity of the inferior avian sensory epithelium following noise exposure (Girod et al., 1989). Corwin. (1981a) also found evidence that HyCs and CCs could translocate, proliferate (not verified here), or directly trans-differentiate into new HCs.

#### 4.1.2 GJs and TCC—Corridors of Gene Transfer?

Crocodile SHCs and THCs were surrounded by a mosaic of electron-lucent SCs coupled with an elaborate system of GJs. In the mammalian ear, GJs are involved in the generation of the endo-cochlear potential and K^+^ recycling, and they are crucial for human hearing (Kelsell et al., 1997; Wangemann, 2002). GJs contain connexin proteins important for intercellular Ca^2+^ signaling, metabolic supply, and fluid homeostasis (Hibino and Kurachi, 2006). In the chicken ear, screening showed two isolates of a Connexin31 cDNA confined to the basilar papilla in SCs, tegmentum vasculosum, CCs, and clear cells (Heller et al., 1998). We tested Cx26 and Cx30 antibody labeling, but found no expression. More analyses, including Cx31 and pannexin, seem necessary. The widespread and designated distribution of GJs in the crocodile hearing organ may suggest that they are dynamic and can enlarge, adapt, and permit even larger-scale intercellular communication. GJs form direct channels between cells, allowing passage of small non-coding miRNAs between cells. They may regulate intercellular genetic communication and play a role in the differentiation of cells during organ development (Zhu et al., 2015; Zong et al., 2016). GJ proteins have been shown to have a remarkably short half-life of only a few hours (Laird, 2006), thus suggesting that a rapid synthesis is essential to uphold their function. GJs can be remodeled by insults (Dhein and Salameh, 2021), and gene regulation may be upregulated with increased protein levels and altered localization, depending on the physiological demands (Risek et al., 1990). GJs could therefore play a crucial role in coordinating the rapid regeneration of HCs observed in avians where new HCs appear after only 4–5 days following damage (Benkafadar et al., 2021). We found that the TCC micro-tubules had a diameter of approximately 20 nm and could allocate larger molecules across cell borders accompanied by cooperative GJs. In the crocodile, both efferent nerves and SCs facing the SHCs were found to contain TCC organelles. Instruments that regulate the cellular movement of small RNAs are largely unknown, but it has been suggested that micro-tubule dynamics could regulate miRNA directional activity locally as well as cell-to-cell movement at some cell interfaces (Skopelitis et al., 2018; Fan et al., 2022).

Similar but slightly different assemblies have been described by other authors. HyCs in the avian organ were labeled with single or aggregates of striated rough tubular elements (94 nm diameter) (Oesterle et al., 1992). These were up to 3–4 µm in length and 0.5 µm in diameter and consisted of stacks of up to 31 tubules arranged in parallel. Cross-sectional analysis revealed that some contained amorphous material, often contiguous with a rough-surfaced endoplasmic reticulum. Anatomically, similar hollow tubules of about 180 A in diameter were also described in the ventricular muscle of toads and opossum testicular interstitial cells (Christensen and Fawcett, 1961; Nayler and Merrillees, 1964). Tubular structures were loosely packed in parallel with undulations, giving rise to transversely cut profiles often near the cell nucleus but without direct connection. Bundles of dense tubules were also seen in HCs in the chicken basilar papilla with dimensions similar to micro-tubules, but these had thicker walls and were not as straight as micro-tubules (Tanaka and Smith, 1978). Their function remains unknown.

#### 4.1.3 What Factors Trigger HC Regeneration?

To create “a crocodile regenerative state” in the human cochlea, it is essential to explore what triggers the SCs to form new HCs. Several genes have been associated with regenerative proliferation in avian SCs. Gene expression changes were analyzed during HC degeneration and apoptosis (Benkafadar et al., 2021). RNA screens of transcription factor genes have identified pathways necessary for sensory regeneration in the avian ear, such as WNT, PAX, and AP1 pathways (Alvarado et al., 2011). Also, different putative HC-specific genes were identified within the NOTCH signaling system where HES7 was expressed during utricle HC regeneration. The expression of the essential ATOH1 gene was closely related to HEYL and HLH inhibitory transcription factors ID1, ID2, and ID4 (Ku et al., 2014). Transcriptome datasets were generated to identify critical genes and molecular pathways (Janesick et al., 2021). Single-cell RNA sequencing and specific markers, typically of SHCs, THCs, and a new subgroup of THCs, were identified. Moreover, markers for SCs or stem cells associated with THCs and SHCs were recognized (Janesick et al., 2021). *In vitro* experiments were also performed and showed that reparation is influenced by soluble elements, factors that have not yet been identified (Tsue et al., 1994). Notably, we found typically secretory-like vesicles restricted to areas where HCs seemed to form; thus, further chemical analyses are necessary.

#### 4.1.4 Can HC Regeneration Be Triggered by Efferent Nerves?

**FIGURE 15:**
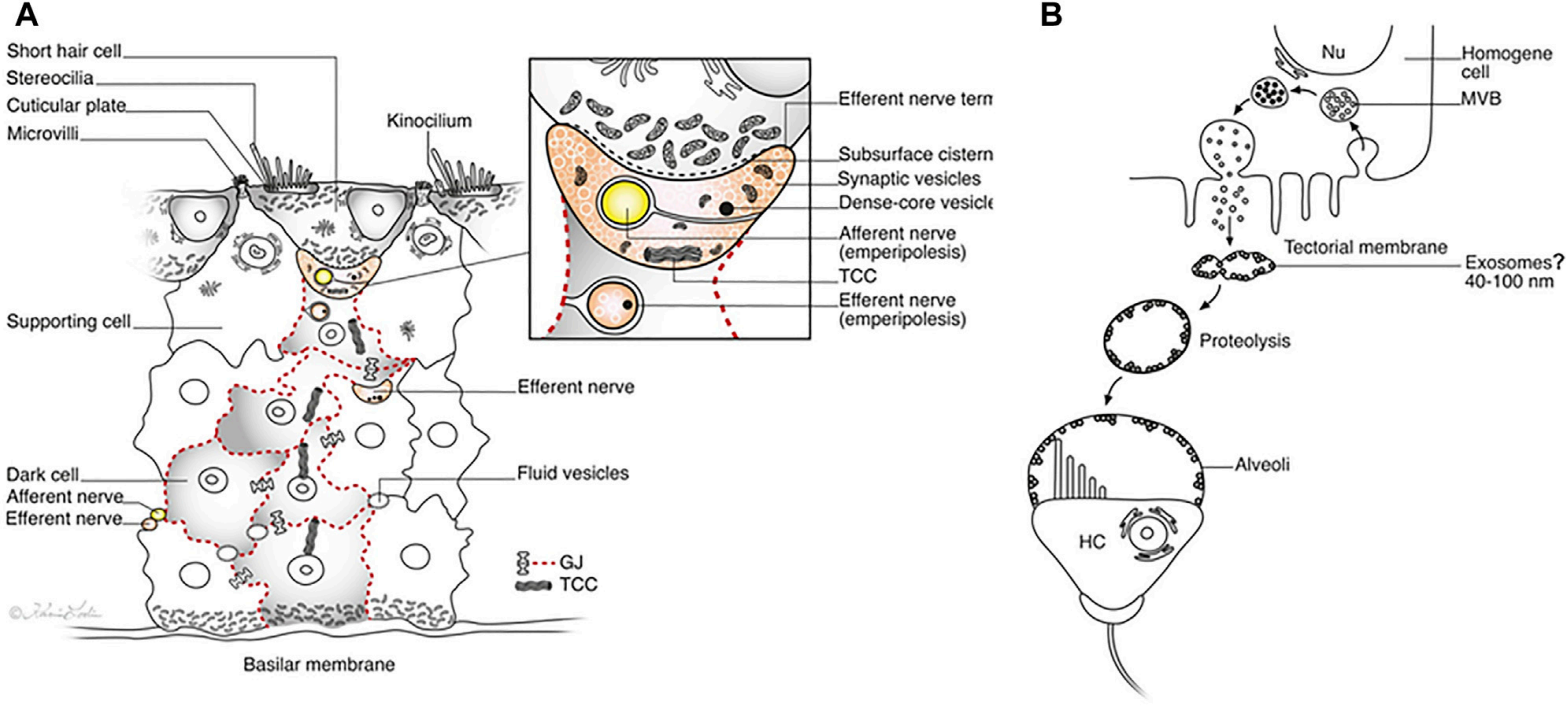
**(A)**. Illustration showing the proposed steps in the development and re-innervation of HCs in the crocodile papilla basilaris. Activated clusters of electron-dense SCs are margined by GJs (red broken lines). The efferent terminal is encircled by an afferent neuron (emperipolesis), while the SC enfolds an efferent neuron. Intercellular communication and the transfer of genetic information between cells may be gated by GJs and the TCC. The inset shows the synaptic region at higher magnification. **(B)**. Formation of HC alveoli. Endocytosis of microvesicles and formation of MVBs is followed by secretion of granules and shedding of exosome-like structures. They appear to have a lytic action of the TM matrix, leading to the formation of clear cavities or alveoli around the HC cilia.

The present study postulates that the crocodile could mediate apoptotic signals from wasted HCs to trigger SCs to form new receptors via the efferent nerve system. In the crocodile, both types of HCs are innervated by afferents and efferent nerves. Efferent terminals were particularly impressive on the SHCs. However, the functional significance of the morphological separation of HCs in crocodile auditory organs remains undetermined. The solid mosaic of SCs seems to restrict the motility of HCs and partly exclude a somatic prestin-based cochlear amplifier that is present in mammals (Dallos, 1992; Zheng et al., 2000). Likewise, chick HCs did not exhibit somatic electro-motility by direct measurements of voltage-dependent length changes in both THSs and SHCs (He et al., 2003). There is also a low density of motor proteins in the in the non-mammalian HCs refuting the idea of a somatic motility (Köppl et al., 2004). Hence, frequency tuning in crocodile HCs may, as in the avian basilar papilla, stem mostly from electrical resonance (Fuchs et al., 1988; Tan et al., 2013; Xia et al., 2016). SLC26A5 mRNA was found infrequently in chicken HCs, and the gene was not differentially enriched in SHCs (Janesick et al., 2021). Nonetheless, a cochlear amplifier was alleged to be present among amniotes (Manley, 2000; Stewart and Hudspeth, 2000), and immune-labeling in chickens SHCs suggested that they may possess an electromechanical force generator and active hair bundle motion (Beurg et al., 2013). Oto-acoustic emissions registered in the chicken ear were presumed to derive from electrically evoked stereocilia bundle movements (Chen et al., 2001). The present study determined for the first time that both THCs and SHCs in the crocodile express SLC26A5/prestin. This also included a few subjacent cells conceivably representing immature HCs that had not yet reached the epithelial surface. In mammals, autonomic nerves may modulate peripheral auditory input. A similar physiological requisite for imposing an efferent supply in the crocodile papilla seems less likely. Subsurface lateral cisternae were not present in the HCs. The prominent efferent nerves and the regulated HC motility may suggest that these nerves serve additional purposes. It was suggested, somewhat controversially, that centrally originating efferent fibers in the mammalian cochlear and vestibular nerves are parasympathetic (Ross, 1969; Ross and Jones, 1981). The special relationship between efferent nerves and transforming SCs in the crocodile auditory organ, including emperipolesis, could suggest that these nerves play an important role in HC renewal (Figure 15A). Tissue regeneration following damage is known to depend on the peripheral nerves in many non-mammalians. Parasympathetic innervation has been shown to be important for the regeneration of several tissues to restore function after damage (Knox et al., 2013). A similar function could serve to replenish HCs in the crocodile auditory epithelium, organization, and innervation. Their role during regeneration also raises intriguing possibilities that HCs could be restored with nerve stem cell therapy and the support of parasympathetic nerves, including the neurotrophic factor neurturin. Neurturin is a member of the glial cell-derived neurotrophic factor (GDNF) family of ligands, which is essential for the development of cranial parasympathetic ganglion neurons (Kotzbauer et al., 1996). Results have shown that cholinergic innervation may even be essential to preserving the structural integrity of certain epithelia (Wanigasekara et al., 2004).

The efferent cochlear bundle was described in the caiman (South American alligator). The efferent fibers that continue beyond the apical end of the cochlea are known to terminate in the macula lagenae. The organization is phylogenetically similar to that in mammals, including man (Rasmussen, 1953; Gacek and Rasmussen, 1961; Rask-Andersen et al., 2000; Liu et al., 2020). Their role is generally believed to be inhibitory, but their wider function in non-mammalian hearing organs is unknown (Köppl, 2022). The present findings may support the notion that the efferent innervation in the crocodile auditory organ could direct the refurbishment of HCs from apoptotic signaling. During the stepwise differentiation of SCs, efferent nerve fibers derived from VCA could monitor and organize the maturation of cells and their innervation. The efferent nerve fibers increased in size and seemed to “guide” cells to the sensory layer and vice versa through physical and molecular interaction (emperipolesis). Several nerve fibers were embedded in SCs and sealed by GJs. Large efferent nerves with multiple synaptic vesicles and mitochondria were closely associated with TCCs during the regenerative process. The efferent neurons also encircled afferents to direct them to the HCs. These findings could suggest that the efferent nerves play a role in the regeneration of new HCs as well as the regulation of their afferent innervation.

### 4.2 Regeneration of the TM

As in the avian, the crocodile auditory papilla is covered by a TM consisting of an extracellular matrix with fine filaments and non-collagenous proteins (Tanaka and Smith, 1975; Killick et al., 1992; Killick et al., 1995). The formation and regeneration of the TM have been studied in the avian papilla (Cotanche, 1987; Goodyear and Richardson, 2018). The amorphous component is believed to be secreted from the HoCs (Cohen and Fermin, 1985; Shiel and Cotanche 1990), while the lower columnar matrix is produced by the SCs. The two components are separated by a longitudinal ridge (Shiel and Cotanche, 1990). However, the mRNA transcripts for TECTA and TECTB were not present in the HoCs during development or in adults, while *β*-tectorin mRNA was expressed in the clear cells, SCs, and CCs (Heller et al., 1998; Coutinho et al., 1999). After damage, the TM showed signs of regeneration from the papilla, but not the HoCs (Cotanche, 1987). The present study indicates that the main part of the TM may be produced by HoCs. There were signs of merocrine and apocrine secretion. These cells were highly differentiated, with prominent finger-like microvilli, coated pits, and microvesicles (MVs). MVBs were present in the HoCs, but also in the HCs.

#### 4.2.1 Can Exosomes Sculpt the Extracellular Matrix of the TM in Crocodiles?

In all crocodiles, the lower surface of the TM showed hollow spaces, or alveoli. These honeycomb-like cavities formed cups containing endolymph around the ciliary tufts. This condition could reduce the viscous damping of the stereocilia and increase receptor sensitivity. In both crocodiles investigated using TEM, the columnar HoCs contained vesicles filled with electron-dense granules that seemed to be expelled into the lumen. Clear spaces developed around the granules in the TM matrix that increased in size against the HCs and in the lateral region. This was especially true near the sensory epithelium. The clear spaces were outlined by membrane-bound exosome-like figures having a diameter of less than 100 nm. These vesicles had a hollow and less electron-dense interior, and occasionally they appeared inside larger membrane-bound EVs in the TM seemingly derived from an MVB (Supplementary image 4). The figures had a lytic influence on the amorphous substance of the TM, a function already postulated by Dohlman (1970). He discovered small grain-like dots in the compact ground substance in the pigeon under an electron microscope and assumed they were residual lysosomes containing keratolytic enzymes. He suggested that there was an “inside” digestive process of enzymes expelled from the “long slender cells” (HoCs) or the light cells in tegmentum vasculosum. It is known that exosome-associated matrix metalloproteinases (MMPs) are known to cleave proteins and remodel the extracellular matrix (Mohan et al., 2016; Shimoda and Khokha 2017). EVs may contain proteins, RNA transcripts, microRNAs, and DNA, including cell surface-bound and soluble matrix MMPs. EV-associated MMP can target cells but also directly degrade the extracellular matrix. In the present investigation, electron microscopy showed 50–100 nm-sized hollow membrane-bound structures together with smaller electron-dense components outlining the TM alveoli in all ears. In the crocodile TM, enzymatic activity by exosomes and their derivatives could profile the alveoli and shape their final architecture. Through them, crocodile could have found a way to increase the sensitivity of the HCs by reducing mechanic ciliary resistance, compensating for restricted electro-motility (Figure 15B). Further analyses of the expression of characteristic biomarkers, such as certain tetraspanin proteins highly enriched in exosomes (CD81, CD82, CD37, and CD63) (Pegtel and Gould, 2019), and involved endopeptidases are needed to fully understand the biology and function of these structures.

## 5 Conclusion

Crocodilians seem to produce new HCs throughout their lives. The auditory organ contains noticeable efferent nerve fibers derived from the vestibulocochlear anastomosis, whose function remains undefined. We postulate that these efferent nerve fibers may play a role in the regeneration and afferent re-innervation of the crocodile auditory receptors, possibly triggered by apoptotic signals from wasted HCs. Neural emperipolesis, elaborate GJs, and specialized transcellular organelles may constitute important gateways for intercellular signaling. These reptiles may also possess intriguing abilities to restore and sculpt the TM matrix through exosome-like proteolysis. We hypothesize that the formed alveolar spaces could reduce ciliary viscous damping and improve the sensitivity of the acoustic receptors.

## Data Availability

The original contributions presented in the study are included in the article/Supplementary Material further inquiries can be directed to the corresponding author.
