# Supplementary figures and images for: Regeneration in the Auditory Organ in Cuban and African Dwarf Crocodiles (Crocodylus rhombifer and Osteolaemus tetraspis) Can We Learn From the Crocodile How to Restore Our Hearing?
Source: Front Cell Dev Biol. 2022 Jul 4;10:934571. doi: 10.3389/fcell.2022.934571 (PMC9289536; doi:10.3389/fcell.2022.934571)

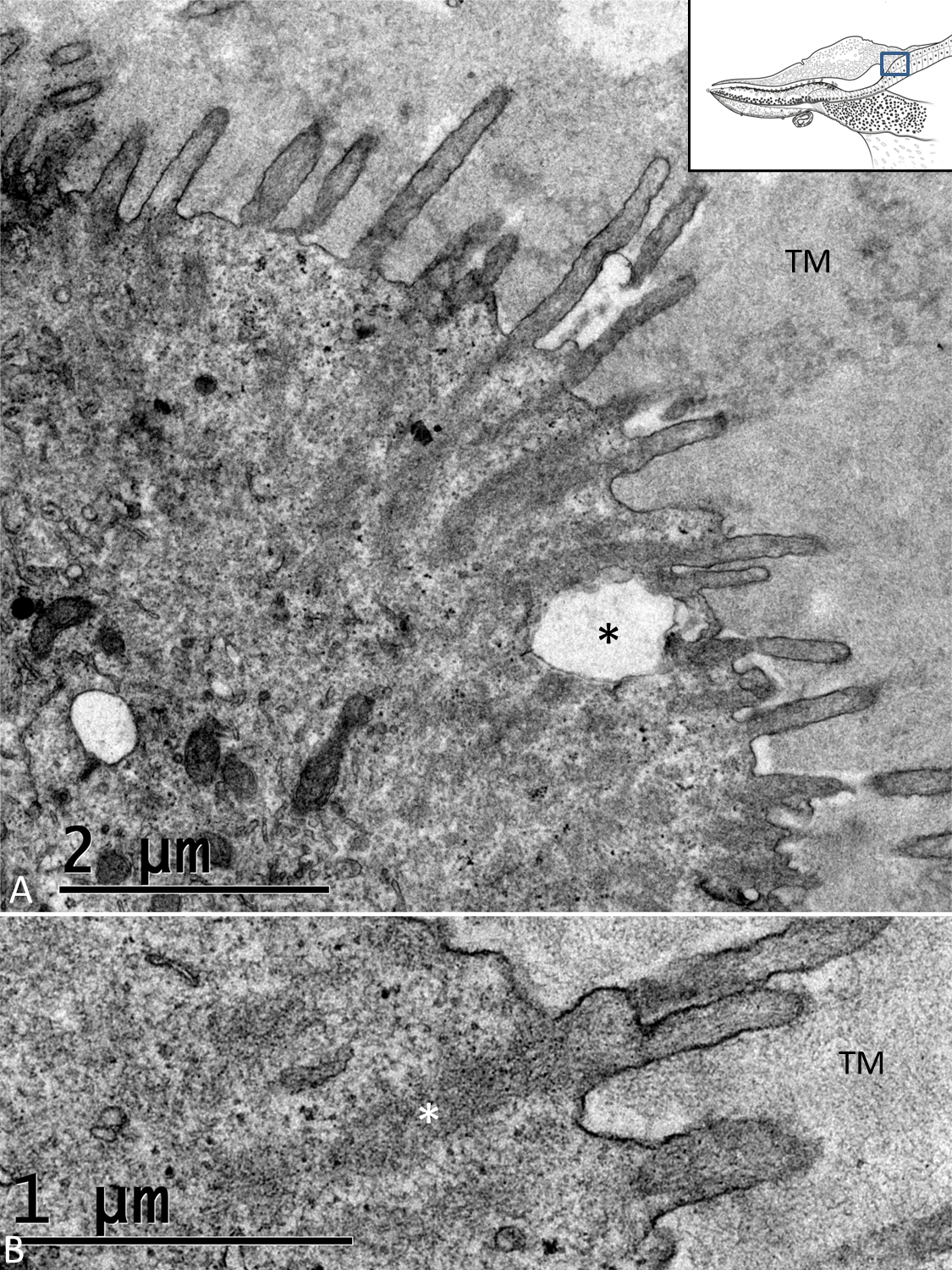

Supplement: Supplementary file 1 [file Image3.TIFF]

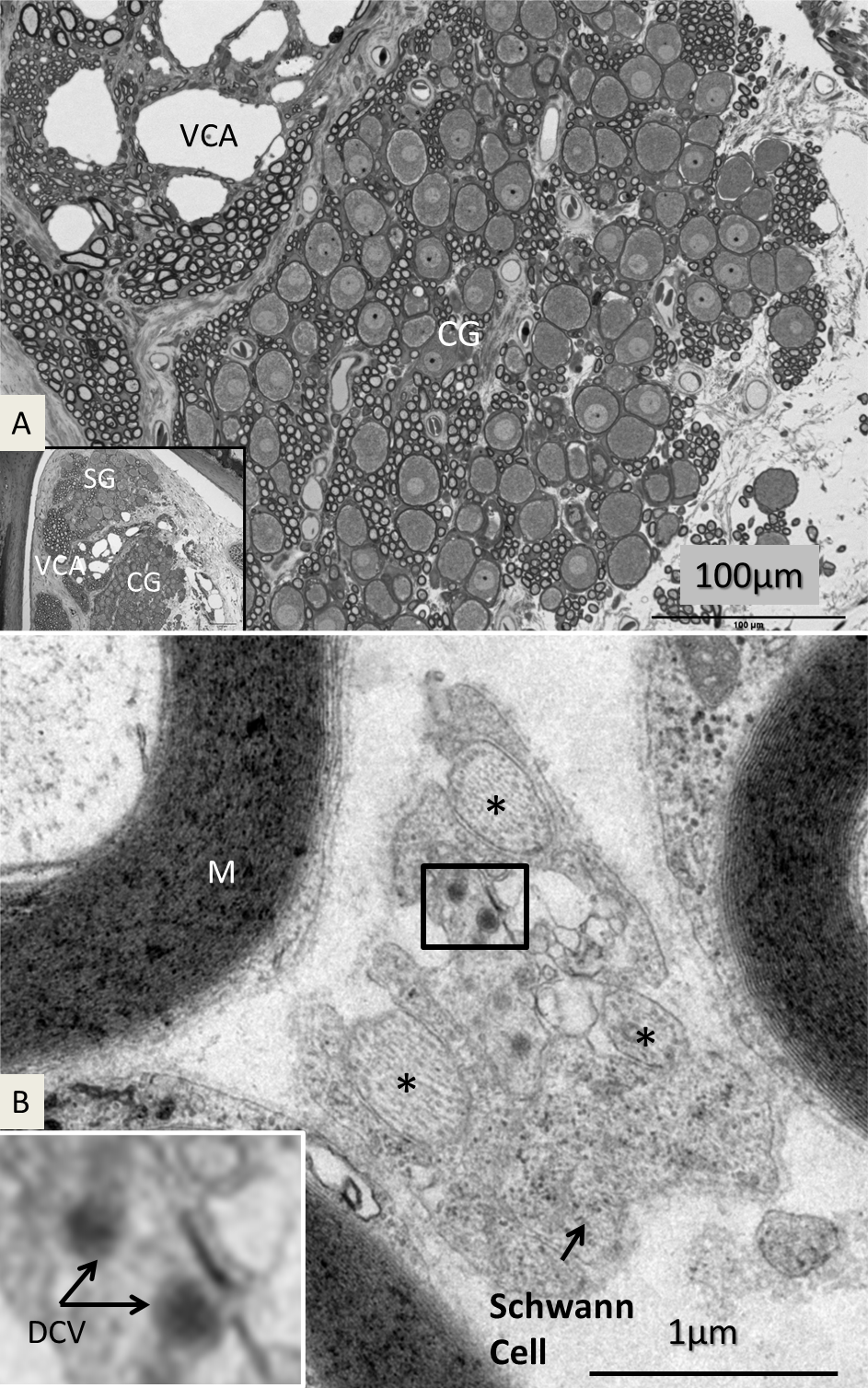

Supplement: Supplementary file 2 [file Image1.TIFF]

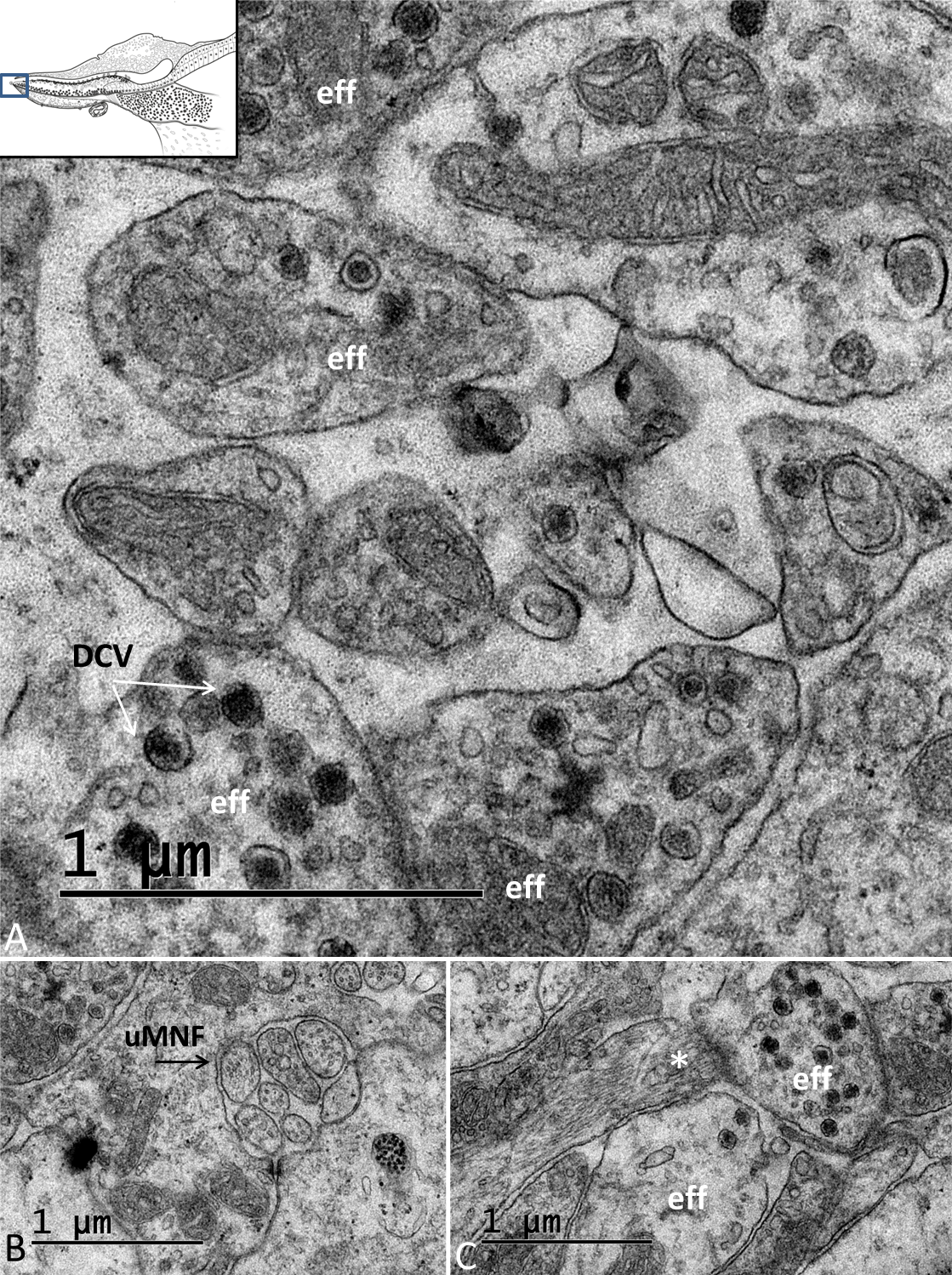

Supplement: Supplementary file 3 [file Image2.TIFF]

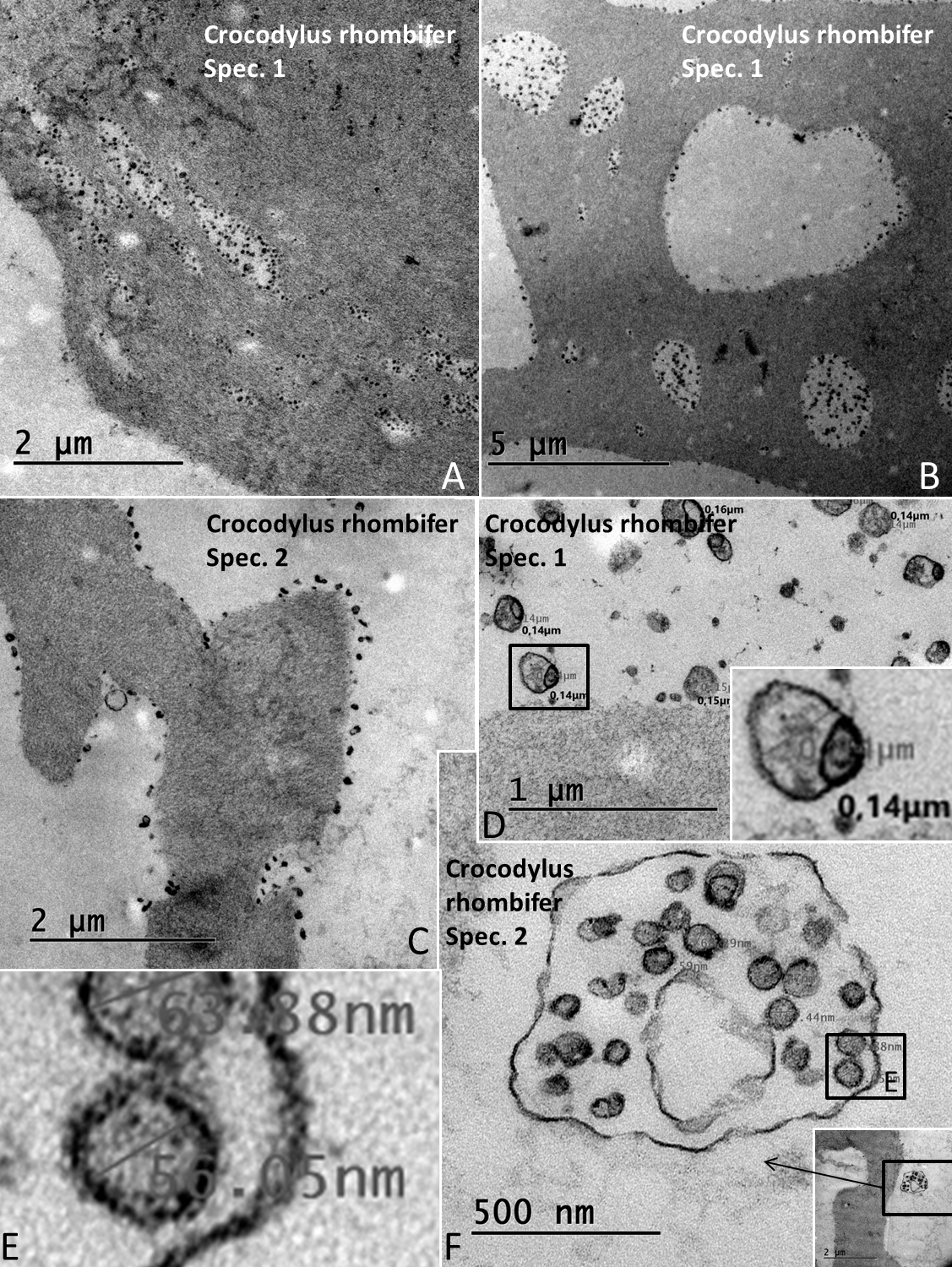

Supplement: Supplementary file 4 [file Image4.TIFF]
